# *Inocybaceae* of Pakistan: a checklist and description of two novel *Inocybe* species using morphological and multi-locus phylogenetic evidence

**DOI:** 10.3897/mycokeys.131.179438

**Published:** 2026-04-17

**Authors:** Sana, Munazza Kiran, Frazana Rafique, Irina Druzhinina, Vasco Fachada

**Affiliations:** 1 Department of Botany, Division of Science & Technology, University of Education, Lahore, 54500, Pakistan Royal Botanic Gardens, Kew London United Kingdom; 2 Royal Botanic Gardens, Kew, Richmond, Surrey, London TW9 3AE, UK Department of Botany, Division of Science & Technology, University of Education Lahore Pakistan

**Keywords:** *
Agaricales
*, *
Basidiomycota
*, fungal diversity, new species, South Asia

## Abstract

The family *Inocybaceae* (*Agaricales*, *Basidiomycota*) comprises ecologically important ectomycorrhizal fungi widely distributed across temperate and tropical forests. Pakistan hosts a diverse representation of this family, particularly species of the genus *Inocybe*. This study presents a comprehensive checklist of members of the family *Inocybaceae* reported from Pakistan. Based on the latest published data, the country contains 55 species of *Inocybaceae*, including 28 of the genus *Inocybe*, six of *Inosperma*, ten of *Mallocybe*, and 11 of *Pseudosperma*, while no records of *Auritella*, *Nothocybe*, or *Tubariomyces* have been documented in the country to date. Additionally, two new species, *Inocybe khalidii* and *Inocybe floribundae*, are described herein based on integrated morphological and multigene phylogenetic analyses. Phylogenetically, *I. khalidii* forms a distinct and strongly supported clade within the genus, confirming its relationship to allopatric species from Mexico, Japan, and the USA, while *I. floribundae* forms a well-supported clade within the Xanthomelas group, sister to European *I. corsica* and *I. diabolica*. Basidiomata of *I. khalidii* are differentially characterized by a vivid orange-brown subglabrous pileus, large and broadly ellipsoid to ellipsoid basidiospores, and a near absence of caulocystidia. Basidiomata of *I. floribundae* are morphologically distinct from their phylogenetic relatives by the beige to brown, distinctly scaly pileus, bulbous but weakly marginate stipe, apically restricted caulocystidia, utriform-obclavate hymenial cystidia, and subisodiametric, bluntly nodulose basidiospores. Morphological descriptions, illustrations, micrographs, and sequences of ITS+LSU- and *tef*1-based phylogenetic analyses are presented in this article with reference to the newly described species.

## Introduction

The family *Inocybaceae* Jülich (*Agaricales*, *Basidiomycota*) is an important component of forest ecosystems, forming ectomycorrhizal (ECM) associations predominantly with plant species from the families *Fagaceae* Dumort., *Pinaceae* Lindl., and *Salicaceae* Mirb. (Kirk et al. 2008), *Cistaceae* Juss. (Fachada et al. 2024), and others. Previously, *Inocybaceae* comprised only three genera: *Auritella* Matheny & Bougher, *Inocybe* (Fr.) Fr., and *Tubariomyces* Esteve-Rav. & Matheny. However, later, Matheny et al. (2020) elevated two former subgenera of *Inocybe*, i.e., subg. *Inosperma* Kühner and subg. *Mallocybe* (Kuyper) Vizzini, Maggiora, Tolaini & Ercole, to the generic rank within *Inocybaceae*. They also described *Nothocybe* Matheny & K.P.D. Latha and *Pseudosperma* Matheny & Esteve-Rav. as new genera within the family. Currently, *Inocybaceae* comprises seven genera: *Auritella*, *Inocybe* sensu stricto (s.s.), *Inosperma*, *Mallocybe*, *Nothocybe*, *Pseudosperma*, and *Tubariomyces*.

*Inocybe* s.s., typified by *I. relicina* (Fr.) Quél., is considered the most diverse and largest genus in the family *Inocybaceae*. Microscopically, members of this genus are characterized by the presence of metuloid cystidia (mostly thick-walled and crystalliferous), hyaline basidia lacking necropigment, and variably shaped basidiospores, with outlines ranging from elliptic to amygdaliform or angular to nodulose or spinose (Matheny et al. 2020). Currently, the genus contains ca. 1,000 accepted species (Pošta et al. 2023), but estimates suggest that the number could reach 3,000–5,000 within *Inocybe* s.s. alone (Bhunjun et al. 2022). This highlights the need for continuous efforts to formally and adequately describe more species of *Inocybe* s.s. (Fachada et al. 2024).

Pakistan is characterized by a tropical to temperate climate, ranging from hot deserts to Himalayan peaks with cold climates. This supports a high diversity of macrofungi, including the family *Inocybaceae* (Khan 2019). Although earlier works (Ahmad et al. 1997; Sultana et al. 2011; Khalid 2022; Aman et al. 2022) attempted to summarize the mycobiota of Pakistan, these compilations covered broader *Basidiomycota* and did not provide a taxonomically updated or family-focused treatment of *Inocybaceae*. Recently, many *Inocybaceae* species have been reported (Hanif et al. 2022; Saba et al. 2020; Crous et al. 2023; Naseer et al. 2023a, 2023b; Razzaq et al. 2023; Saba et al. 2023; Naseer et al. 2024; Ashraf et al. 2025; Faryad et al. 2025; Sana et al. 2025a, 2025b; Ahmad et al. 2025) from the country based on molecular phylogenetic studies. Moreover, several previously reported taxa no longer retain their original taxonomic placement, having been reassigned at the genus or family level. Therefore, a dedicated, up-to-date checklist of *Inocybaceae* remains lacking, and this study aims to address this gap. Here, an effort has been made to list all the species that have been legitimately published and to exclude those that have been invalidly published and listed. In total, 55 valid species were recorded, along with their localities, habitats, herbarium vouchers, GenBank accession numbers, MycoBank/IF numbers, and dates of taxon discovery. Additionally, to further explore the diversity of the family *Inocybaceae* in Pakistan, various mycological surveys were conducted during the monsoon seasons of 2023 and 2024 across various localities in Swat, Khyber Pakhtunkhwa (KP), Pakistan, resulting in the discovery of two previously undocumented species of *Inocybe*. Detailed morpho-anatomical characterization, together with rRNA gene cluster (ITS+LSU) and *tef*1-based phylogenetic analyses, confirmed that the collected species are novel.

## Materials and methods

### Sampling site

Basidiomata were collected from *Quercus* L.-dominated forests around Shawar Valley and Aligrama Village, Swat District (34.34–35.55°N, 72.08–72.50°E), KP Province, Pakistan, during field surveys conducted in the monsoon seasons of 2023 and 2024. Swat is characterized by a variety of phytogeographical regions, intense monsoonal precipitation, a humid subtropical climate, and high biodiversity (Ali and Qaiser 1986). Alpine pastures, meadows, and cold desert dominate the northern regions of Swat; in contrast, the southern regions are primarily characterized by subtropical chir pine forests, providing a diverse and optimal habitat for macrofungi (Ahmad et al. 2015).

### Morpho-anatomical characterization

Basidiomata of *Inocybe* were photographed in their natural habitat, tagged with collection details (Rathnayaka et al. 2025), placed in plastic collection boxes, and taken to the mycology laboratory at the University of Education, Lahore. Morphological characteristics were recorded from fresh specimens according to Vellinga (2001). Colors were designated with reference to the Munsell color chart (1975). Illustrations were produced after tuning four iterations of a large language model (OpenAI 2025) by prompting detailed written taxonomic descriptions and uploading a minimum of two field photographs per species. Collections of the newly described species were deposited in the Lahore Herbarium at the University of the Punjab, Lahore, Pakistan (herbarium code LAH). Microscopic characters are based on freehand sections from fresh and dried specimens mounted in 5% (w/v) aqueous potassium hydroxide (KOH) solution. Congo red (1% w/v) was used to stain the tissues, followed by examination using a compound microscope (S/N-EU 2230435) at 40× (N/A = 0.65) and 100× (N/A = 1.25) objectives and a 10× eyepiece (Rafique et al. 2025). A total of 20 basidiospores, basidia, cystidia, and hyphae from pilei and stipites were measured from each collection. The abbreviation [n/b/p] refers to the number of basidiospores (n) calculated from the given number of specimens (b) and the number of collections (p). The dimensions of the basidiospores, basidia, and hymenial cystidia are provided according to the equation (a–)b–c(–d); extreme values are given in parentheses, and the range b–c represents a minimum of 90% of the measured values. Basidiospore dimensions are measured as length × width (L × W), with extreme values provided in parentheses. Q = L/W ratio of individual spores; Q_av_ = average Q of all spores (Ishaq et al. 2025).

### Molecular study

Genomic DNA was extracted from portions of lamellae following a modified CTAB extraction method (Gardes and Bruns 1993). Primer pairs used for amplification of the ITS region were ITS1F and ITS4; for the LSU, LR0R and LR5; and for *tef*1, EF1-983 and EF1-1567 (White et al. 1990; Gardes and Bruns 1993; Rehner and Buckley 2005). PCR was performed using a 10 μL reaction volume composed of 5 μL Biomed 2× Taq Plus PCR MasterMix (Biomed, Beijing, China), 3.9 μL ddH_2_O, 0.3 μL of each primer (10 μmol/L), and 0.5 μL DNA template (Khan et al. 2025). PCR products were commercially sequenced at TsingKe, China, using the same primer pairs. Newly generated sequences were submitted to GenBank, and short descriptions were deposited in MycoBank.

### Sequence alignment and phylogenetic analyses

**Figure 1.**
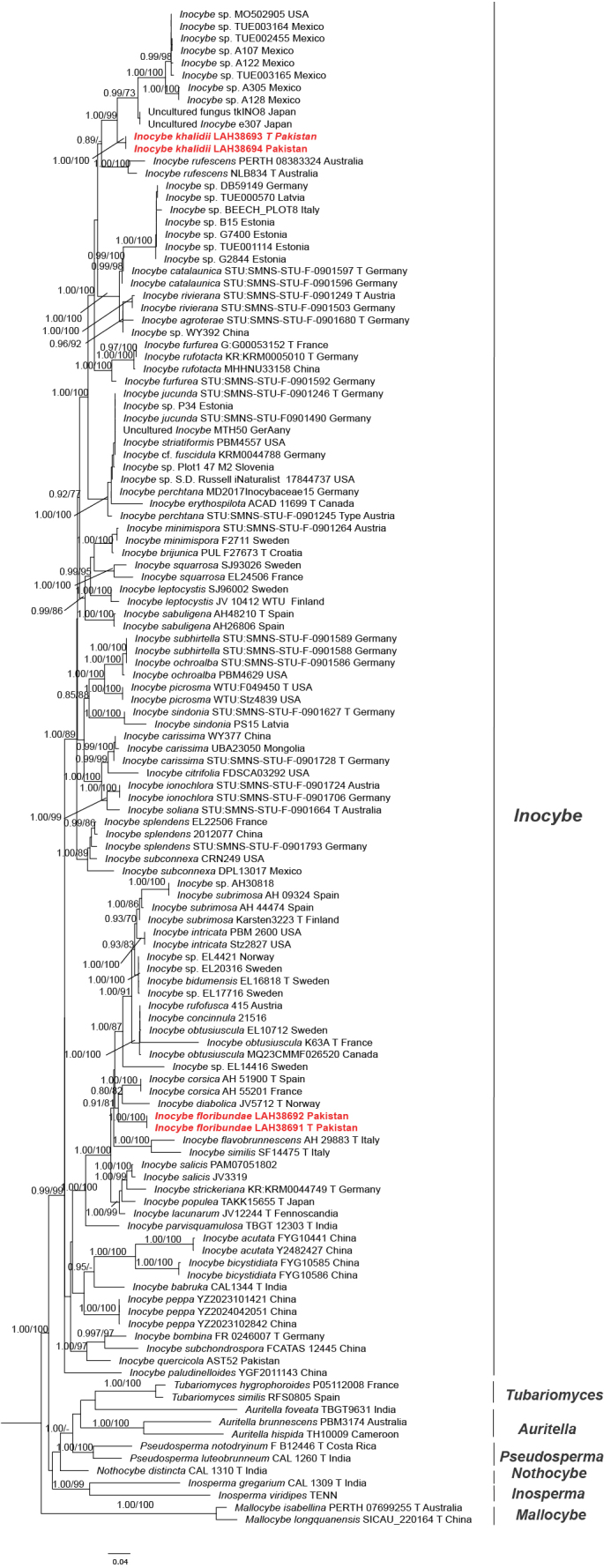
Molecular phylogeny of *Inocybe*, including *Inocybe khalidii* sp. nov. and *Inocybe floribundae* sp. nov., based on maximum likelihood (ML) and posterior probability (PP) analyses of ITS and LSU sequences. Newly generated sequences are indicated in red. The letter “T” before the country name represents sequences from type specimens.

**Figure 2.**
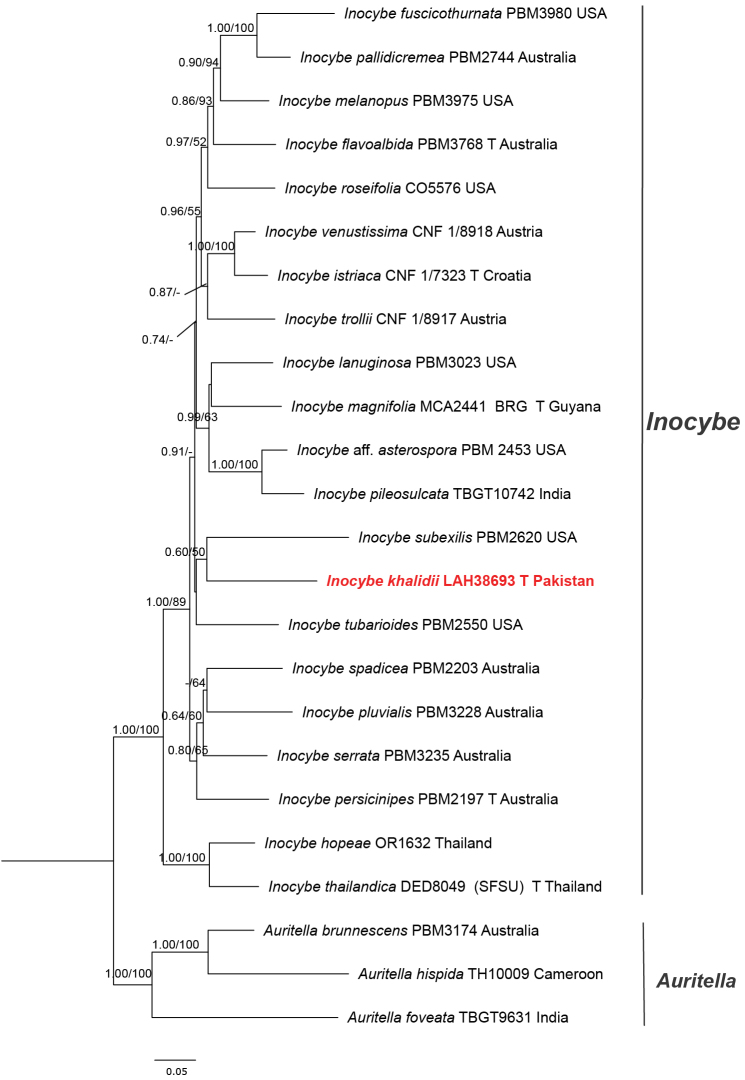
Molecular phylogeny of *Inocybe*, including *Inocybe khalidii* sp. nov., based on maximum likelihood (ML) and posterior probability (PP) analyses of *tef*1 sequences. Newly generated sequences are indicated in red. The letter “T” before the country name represents sequences from type specimens.

BioEdit version 7.0.5.3 was used to assemble nucleotide sequences (Hall 1999). Sequences were compared using the Basic Local Alignment Search Tool (BLAST) at the National Center for Biotechnology Information (NCBI; USA). Additionally, we included all sequences belonging to the same UNITE 1% species hypothesis (SH1207925.10FU-ITS and SH1207924.10FU-LSU for one taxon and SH1212724.10FU-ITS and SH1212762.10FU-LSU for the other taxon) following Fachada et al. (2024). To reconstruct the ITS+LSU- and *tef*-1-based phylogeny, similar sequences were obtained from GenBank and UNITE. Additionally, sister taxa of the species identified in BLAST searches were retrieved from relevant publications on *Inocybe* and incorporated into the final dataset (Bandini et al. 2020a, 2023). Sequences from other genera of *Inocybaceae* were used as outgroups. The final dataset was aligned using the MAFFT online service (https://mafft.cbrc.jp/alignment/server/); the alignments were then manually adjusted in BioEdit version 7.0.9.0 (Hall 1999). Maximum likelihood (ML) analysis was carried out using RAxML HPC2 v. 8.2.12 on the XSEDE tool (8.2.10) implemented on the CIPRES Science Gateway (Miller et al. 2010). The GTR+GAMMA nucleotide substitution model was used. ITS and LSU were concatenated and analyzed together, while *tef*1 was analyzed separately. 1000 bootstrap iterations were performed with rapid bootstrapping. Bayesian inference (BI) analysis was performed using MrBayes v. 3.2.1 (Ronquist et al. 2012). The model of evolution was estimated by MrModeltest 2.2 (Nylander 2004). Markov chain Monte Carlo (MCMC) sampling in MrBayes v. 3.2.2 (Ronquist et al. 2012) was used to estimate posterior probabilities (PPs). Four simultaneous Markov chains were run for 2,000,000 generations, and trees were sampled every 1000^th^ generation. Phylograms were visualized in FigTree v. 1.4.2 (Rambaut 2014). Posterior probability (PP) ≥ 0.50/bootstrap values (BP) ≥ 50% were considered supported and given as branch labels of phylograms (Figs 1, 2 and Table 1). The alignments are available in TreeBASE (www.treebase.org/treebase-web/home.html) under ID 32295 for ITS, LSU, and *tef*1 sequences.

**Table 1.** Taxon name, voucher/strain No., country, GenBank accessions, and references of sequences used in the phylogenetic analyses. The new species is in bold. “T” stands for sequences from type specimens while “-“ represents data unavailable.

Taxon Name	Voucher/Strain No./Reference/Source	Country	GenBank accessions			References
			ITS	LSU	*tef1*	
*Auritella brunnescens*	PBM3174	Australia	KJ702344	JQ313571	MK426176	Matheny and Bougher (2017)
*Auritella foveata*	TBGT9631	India	GU062740	GU062739	MK426177	Ashraf et al. (2025)
*Auritella hispida*	TH10009	Cameroon	KT378203	KT378207	MK426179	Matheny et al. (2017)
*Inocybe acutata*	FYG10441	China	PQ495596	-	-	Unpublished
*Inocybe acutata*	Y2482427	China	PQ495600	-	-	Unpublished
*Inocybe aff. asterospora*	PBM 2453	USA	-	-	DQ435795	Matheny et al. (2007)
*Inocybe agroterae*	STU-SMNS:STU-F-0901680 (T)	Germany	ON003436	In ITS	-	Bandini et al. (2020b)
*Inocybe babruka*	CAL 1344 (T)	India	KY440086	KY549116	-	Latha and Manimohan (2016)
*Inocybe bicystidiata*	FYG10585	China	PQ422907	PQ422909	-	Unpublished
*Inocybe bicystidiata*	FYG10586	China	PQ422908	PQ422910	-	Unpublished
*Inocybe bidumensis*	EL16818 (T)	Sweden	OQ572784	-	-	Crous et al. (2023)
*Inocybe bombina*	FR_0246007 (T)	Germany	NR_173842	-	-	Bandini et al. (2019)
*Inocybe brijunica*	PUL F27673 (T)	Croatia	NR_172782	G_075311	-	Mešić et al. (2021)
*Inocybe carissima*	STU:SMNS-STU-F-0901728 (T)	Germany	OP164087	In ITS	-	Bandini et al. (2022c)
*Inocybe carissima*	WY-377	China	PV412963	PV412972	-	Unpublished
*Inocybe catalaunica*	STU:SMNS-STU-F-0901596	Germany	OK057187	In ITS	-	Bandini et al. (2022a)
*Inocybe catalaunica*	STU:SMNS-STU-F-0901597 (T)	Germany	OK057188	In ITS	-	Bandini et al. (2022a)
*Inocybe cf. fuscidula*	KR-M-0044788	Germany	MT005885	-	-	Unpublished
*Inocybe citrifolia*	FDSCA03292	USA	PQ140069	In ITS	-	Unpublished
*Inocybe concinnula*	21516	-	JF908210	-	-	Unpublished
*Inocybe corsica*	AH 51900 (T)	Spain	NR_176157	NG_088240	-	Crous et al. (2021)
*Inocybe corsica*	AH 55201	France	MZ308645	MZ308647	-	Crous et al. (2021)
*Inocybe diabolica*	JV5712 (T)	Norway	AM882903	-	-	Ryberg et al. (2008)
*Inocybe erythospilota*	ACAD:11699 (T)	Canada	MG489947	In ITS	-	Grund and Stuntz (1984)/ Unpublished
*Inocybe flavoalbida*	PBM3768 (T)	Australia	-	-	MK426183	Matheny et al. (2020)
*Inocybe flavobrunnescens*	AH 29883 (T)	Italy	NR_153145	-	-	Esteve-Raventós et al. (2015)
***Inocybe floribundae***	**LAH38691 (T)**	**Pakistan**	**PX630063**	**PX630065**	**-**	**This Study**
***Inocybe floribundae***	**LAH38692**	**Pakistan**	**PX630064**	**PX630062**	**-**	**This Study**
*Inocybe fuscicothurnata*	PBM3980	USA	-	-	MK426184	Matheny et al. (2020)
*Inocybe furfurea*	STU:SMNS-STU-F-0901592	Germany	OK057169	In ITS	-	Bandini et al. (2022a)
*Inocybe furfurea*	G:G00053152 (T)	France	MG012472	In ITS	-	Bandini et al. (2019)
*Inocybe hopeae*	OR1632	Thailand	-	-	ON553694	Raghoonundon et al. (2023)
*Inocybe intricata*	PBM 2600	USA	EU523561	EU307835	-	Kropp et al. (2010)
*Inocybe intricata*	Stz8287	USA	MH024844	-	-	Unpublished
*Inocybe ionochlora*	STU:SMNS-STU-F-0901724	Austria	OP164054	In ITS	-	Bandini et al. (2022c)
*Inocybe ionochlora*	STU:SMNS-STU-F-0901706	Germany	OP164061	In ITS	-	Bandini et al. (2022c)
*Inocybe istriaca*	CNF 1/7323	Croatia	-	-	OQ596331	Pošta et al. (2023)
*Inocybe jucunda*	STU:SMNS-STU-F-0901246 (T)	Germany	MW578524	In ITS	-	Bandini et al. (2021b)
*Inocybe jucunda*	STU:SMNS-STU-F-0901490	Germany	MW647619	In ITS	-	Bandini et al. (2021b)
***Inocybe khalidii***	**LAH38693 (T)**	**Pakistan**	**PV608030**	**PV608041**	**PV865529**	**This study**
***Inocybe khalidii***	**LAH38694**	**Pakistan**	**PV611493**	**PV639641**	**-**	**This study**
*Inocybe lacunarum*	JV12244 (T)	Fennoscandia	KT958908	-	-	Vauras and Larsson (2016)
*Inocybe lanuginosa*	PBM3023	USA	-	-	MK426186	Matheny et al. (2020)
*Inocybe leptocystis*	SJ96002	Sweden	AM882801	In ITS	-	Ryberg et al. (2008)
*Inocybe leptocystis*	JV_10412[WTU]	Finland	-	AY380384	-	Matheny (2005)
*Inocybe magnifolia*	MCA2441 (BR) (T)	Guyana	-	-	MK426189	Matheny et al. (2020)
*Inocybe melanopus*	PBM3975	USA	-	-	MK426190	Matheny et al. (2020)
*Inocybe minimispora*	STU:SMNSSTUF0901264	Austria	MW845934	In ITS	-	Bandini et al. (2021a)
*Inocybe obtusiuscula*	EL10712	Sweden	OQ572792	-	-	Vauras and Kokkonen (2009)
*Inocybe obtusiuscula*	K63A (T)	France	FJ755800	-	-	Unpublished
*Inocybe obtusiuscula*	MQ23CMMF026520	Canada	PP865766	-	-	Unpublished
*Inocybe minimispora*	F2711	Sweden	PQ652820	In ITS	-	Unpublished
*Inocybe ochroalba*	STU:SMNS-STU-F-0901586	Germany	OK057133	In ITS	-	Bandini et al. (2022a)
*Inocybe ochroalba*	PBM4629	USA	PQ860971	PQ642780	-	Unpublished
*Inocybe pallidicremea*	PBM2744	USA	-	-	MK426191	Matheny et al. (2020)
*Inocybe paludinelloides*	YGF2011143	China	MG938541	-	-	Chen et al. (2024)
*Inocybe parvisquamulosa*	TBGT 12303 (T)	India	NR_185371	KT329453	-	Pradeep et al. (2016)
*Inocybe peppa*	YZ2023101421	China	PQ495598	PQ495604	-	Gao et al. (2025)
*Inocybe peppa*	YZ2023102842	China	PQ495597	PQ495603	-	Gao et al. (2025)
*Inocybe peppa*	YZ2024042051	China	PQ495599	PQ495605	-	Gao et al. (2025)
*Inocybe perchtana*	STU:SMNS-STU-F-0901245 (T)	Austria	MN512326	In ITS	-	Bandini et al. (2020b)
*Inocybe perchtana*	MD2017 *Inocybaceae* 5	Germany	PP695574	In ITS	-	Unpublished
*Inocybe persicinipes*	PBM2197	Australia	-	-	MK426192	Matheny et al. (2020)
*Inocybe picrosma*	WTU:F049450 (T)	USA	NR_121489	-	-	Schoch et al. (2014)
*Inocybe picrosma*	WTU:Stz4839	USA	HQ201364	In ITS	-	Unpublished
*Inocybe pileosulcata*	TBGT10742	India	-	-	MK426193	Matheny et al. (2020)
*Inocybe populea*	TAKK15655 (T)	Japan	KT958911	-	-	Kobayashi and Courtecuisse (2000)
*Inocybe pluvialis*	PBM3228	Australia	-	-	MK426194	Matheny et al. (2020)
*Inocybe quercicola*	AST52	Pakistan	MW412768	-	-	Khan et al. (2021)
*Inocybe rivierana*	STU:SMNS-STU-F-0901249 (T)	Austria	MW845910	In ITS	-	Bandini et al. (2021a)
*Inocybe rivierana*	STU:SMNS-STU-F-0901503	Germany	MW845912	In ITS	-	Bandini et al. (2021a)
*Inocybe roseifolia*	CO5576	USA	-	-	MK426195	Matheny et al. (2020)
*Inocybe rufescens*	PERTH_08383324	Australia	KP308819	KP170992	-	Matheny and Bougher (2017)
*Inocybe rufescens*	NLB834 (T)	Australia	KP308818	KP170991	-	Matheny and Bougher (2017)
*Inocybe rufofusca*	415	Austria	EF655684	-	-	Mühlmann et al. (2008)
*Inocybe rufotacta*	KR:KRM0005010 (T)	Germany	MG012467	In ITS	-	Bandini et al. (2019)
*Inocybe rufotacta*	MHHNU33158	China	PQ756977	In ITS	-	Unpublished
*Inocybe sabuligena*	AH48210 (T)	Spain	PP262648	In ITS	-	Crous et al. (2024)
*Inocybe sabuligena*	AH49106	Spain	PP262650	In ITS	-	Crous et al. (2024)
*Inocybe salicis*	PAM07051802	-	KT958907	-	-	Unpublished
*Inocybe salicis*	JV3319	-	KT958906	-		Unpublished
*Inocybe serrata*	PBM3235	Australia	-	-	MK426197	Matheny et al. (2020)
*Inocybe similis*	SF14475 (T)	Italy	MT704951	-	-	Dovana et al. (2020)
*Inocybe sindonia*	PS15	Latvia	KT182917	In ITS	-	Kļaviņa et al. (2016)
*Inocybe sindonia*	STU:SMNS-STU-F-0901627 (T)	Germany	OK057164	In ITS	-	Bandini et al. (2022a)
*Inocybe soliana*	STU:SMNS-STU-F-0901664 (T)	Austria	ON003425	In ITS	-	Bandini et al. (2022b)
*Inocybe* sp.	EL4421	Norway	OQ572787	-	-	Unpublished
*Inocybe* sp.	EL20316	Sweden	OQ572788	-	-	Unpublished
*Inocybe* sp.	EL17716	Sweden	OQ572785	-	-	Unpublished
*Inocybe* sp.	DB5-9-14-9	Germany	MH366576	-	-	Bandini et al. (2020)
*Inocybe* sp.	G2844	Estonia	UDB0551045	-	-	UNITE
*Inocybe* sp.	G7400	Estonia	UDB0652502	-	-	UNITE
*Inocybe* sp.	TUE000570	Latvia	UDB03646109	-	-	UNITE
*Inocybe* sp.	TUE001114	Estonia	UDB03786356	-	-	UNITE
*Inocybe* sp.	B15	Estonia	UDB0699236	-	-	UNITE
*Inocybe* sp.	BEECH_PLOT8	Italy	UDB0785482	-	-	UNITE
*Inocybe* sp.	AH30818	-	KJ938776	-	-	Unpublished
*Inocybe* sp.	P34	Estonia	AJ534897	In ITS	-	Tedersoo et al. (2003)
*Inocybe* sp.	S.D._Russell_iNaturalist_#_17844737	USA	OM473645	-	-	Unpublished
*Inocybe* sp.	Plot1 47 M2	Slovenia	MW027923	In ITS	-	Mrak et al. (2021)
*Inocybe* sp.	A305	Mexico	PV766554	-	-	Unpublished
*Inocybe* sp.	A128	Mexico	PV766377	-	-	Unpublished
*Inocybe* sp.	MO502905	USA	OR336169	-	-	Unpublished
*Inocybe* sp.	TUE002455	Mexico	UDB01967799	-	-	UNITE
*Inocybe* sp.	TUE003164	Mexico	UDB02097608	-	-	UNITE
*Inocybe* sp.	EL14416	Sweden	OM891091	-	-	Unpublished
*Inocybe* sp.	A107	Mexico	PV766356	-	-	Unpublished
*Inocybe* sp.	TUE003165	Mexico	UDB02097953	-	-	UNITE
*Inocybe* sp.	A122	Mexico	PV766371	-	-	Unpublished
*Inocybe* sp.	WY392	China	-	PV383236	-	Unpublished
*Inocybe splendens*	STU:SMNSSTUF0901793	Germany	OR100705	In ITS	-	Bandini et al. (2023)
*Inocybe splendens*	EL22506	France	FN550911	In ITS	-	Ryberg et al. (2010)
*Inocybe splendens*	2012077	China	-	KU764681	-	Fan and Bau (2017)
*Inocybe spadicea*	PBM2203	Australia	-	-	MK426198	Matheny et al. (2020)
*Inocybe striatiformis*	PBM4557	USA	-	PQ860781	-	Matheny and Kudzma (2019)
*Inocybe strickeriana*	KR_KRM0044749 (T)	Germany	NR_185399	NG_228785	-	Bandini et al. (2019)
*Inocybe subrimosa*	AH09324	Spain	ON259056	-	-	Esteve-Raventós et al. (2022)
*Inocybe subrimosa*	AH44474	Spain	ON259054	In ITS	-	Esteve-Raventós et al. (2022)
*Inocybe subrimosa*	Karsten3223 (T)	Finland	ON227436	-	-	Esteve-Raventós et al. (2022)
*Inocybe subexilis*	PBM2620	USA	-	-	MK426199	Matheny et al. (2020)
*Inocybe subhirtella*	STU:SMNS-STU-F-0901588	Germany	OK057135	In ITS	-	Bandini et al. (2022a)
*Inocybe subhirtella*	STU:SMNS-STU-F-0901589	Germany	OK057136	In ITS	-	Bandini et al. (2022a)
*Inocybe subchondrospora*	FCATAS 12445	China	PV827765	-	-	Unpublished
*Inocybe subconnexa*	CRN249	USA	-	PQ860782	-	Unpublished
*Inocybe subconnexa*	DPL13017	Mexico	PQ839750	In ITS	-	Unpublished
*Inocybe squarrosa*	EL24506	France	FN550924	In ITS	-	Ryberg et al. (2010)
*Inocybe squarrosa*	SJ93026	Sweden	AM882790	In ITS	-	Ryberg et al. (2008)
*Inocybe thailandica*	DED8049 (SFSU) (T)	Thailand	-	-	MK426200	Matheny et al. (2020)
*Inocybe trollii*	CNF 1/8917	Austria	-	-	OQ596333	Pošta et al. (2023)
*Inocybe tubarioides*	PBM2550	Austria	-	-	MK426201	Matheny et al. (2020)
*Inocybe venustissima*	CNF 1/8918	Austria	-	-	OQ596332	Pošta et al. (2023)
Uncultured fungus	tkINO8	Japan	PP922646	-	-	Janowski and Nara (2024)
Uncultured *Inocybe*	e307	Japan	LC367861	In ITS	-	Miyamoto et al. (2018)
Uncultured *Inocybe*	Mth50	Germany	KT020788	-	-	Unpublished
*Inosperma viridipes*	TENN 066999 (T)	Australia	NR_153168	-	-	Matheny and Bougher (2017)
*Inosperma gregarium*	CAL 1309 (T)	India	NR_153174	KX852306	-	Latha and Manimohan (2016)
*Mallocybe isabellina*	PERTH:07699255 (T)	Australia	NR_152356	NG_057248	-	Matheny and Bougher (2017)
*Mallocybe longquanensis*	SICAU_220164 (T)	China	OQ434283	OQ434278	-	Liu et al. (2024)
*Nothocybe distincta*	CAL 1310 (T)	India	NR_173156	NG_057278	-	Latha et al. (2016)
*Pseudosperma notodryinum*	F B12446 (T)	Costa Rica	NR_164070	NG_244400	-	Matheny et al. (2020)
*Pseudosperma luteobrunneum*	CAL 1260 (T)	India	NR_153171	NG_057275	-	Tibpromma et al. (2017)
*Tubariomyces hygrophoroides*	P05112008	France	GU907094	GU907094	-	Alvarado et al. (2010)
*Tubariomyces similis*	RFS0805	Spain	GU907096	GU907092	-	Alvarado et al. (2010)

### Checklist preparation

This checklist is based on the latest published articles and literature searches on the family *Inocybaceae* in Pakistan. The status, names, authorities, and synonyms of the species are given according to the Index Fungorum and Species Fungorum (http://www.indexfungorum.org and http://www.speciesfungorum.org; retrieved on March 27, 2026).

## Results

### Phylogenetic analyses

(Figs 1, 2; Table 1)

Eight sequences (four ITS, three LSU, and one *tef*1) from two different collections were generated and submitted to GenBank. The final ITS and LSU-based combined dataset comprised 124 sequences with 2684 nucleotide sites, 1557 distinct patterns, 823 parsimony-informative sites, and 1557 constant sites, as well as invariant sites, while the *tef*1 dataset contains 22 sequences with 1107 columns, 529 distinct patterns, 362 parsimony-informative sites, 130 singleton sites, and 615 constant sites. Phylogenetic trees inferred from ML and BI analyses were nearly identical in topology; therefore, the optimal ML phylogram with combined support data is presented (Figs 1, 2). In the ITS+LSU-based phylogram, *Inocybe khalidii* (holotype: LAH38693; paratype: LAH38694) and *Inocybe floribundae* (holotype: LAH38691; paratype: LAH38692) formed distinct and well-supported monophyletic groups with maximum support values (PP = 1.00/BP = 100). *Inocybe khalidii* appeared as a sister taxon to a distinct clade of unnamed or uncultured *Inocybe* sequences submitted from Mexico (TUE002455, A107, TUE003165, TUE003164, A305, A128, A122), Japan (tkIN08, e307), and the USA (MO502905), indicating a close evolutionary relationship with significant divergence (PP = 1.00/BP = 99). This clade was nested within a broader lineage that includes other well-supported species such as *I. rufotacta* Schwöbel & Stangl (MHHNU33158 and KR KRM 0005010) from China and Germany, *I. furfurea* Kühner (G: G00053152 and STU: SMNS-STU-F-0901592) from France and Germany, *I. rufescens* Matheny & Bougher (NLB834 and PERTH 08383324) from Australia, *I. agroterae* Bandini & B. Oertel (STU: SMNS-STU-F-0901680) from Germany, *I. catalaunica* Singer (Type SingerIX34 and STU: SMNS-STU-F-0901596) from Spain and Germany, and *I. rivierana* Bandini & B. Oertel (STU: SMNS-STU-F-0901249; STU: SMNS-STU-F-0901503) from Austria and Germany. The phylogenetic tree inferred from the ITS and LSU dataset (Fig. 1) placed *I. floribundae* as a separate group sister to a clade containing European *I. corsica* Esteve-Rav., Pancorbo & G. Moreno (AH 55201 and AH 51900) and *I. diabolica* Vauras (JV5712) from Norway within the Xanthomelas group. Other closely related species to *I. floribundae* are *I. flavobrunnescens* Esteve-Rav., G. Moreno & Bizio (AH29883) and *I. similis* Bres. (SF14475) from Italy. In contrast, *tef*1-based phylogenetic inference placed *I. khalidii* in a separate species-level clade sister to *Inocybe subexilis* (Peck) Sacc. (PBM2620) from the USA with weak support values (PP = 0.60/BP = 50%). We failed to obtain *tef*1 for *I. floribundae* despite multiple attempts. The newly generated taxa are highlighted in red script in the phylograms (Figs 1, 2) and in bold in Table 1. This placement highlights the distinct phylogenetic position of both species within the genus.

### Taxonomy

#### Inocybe khalidii

Taxon classificationFungiAgaricalesInocybaceae

Sana, Fachada & Kiran sp. nov.

B1663FFD-C99B-570F-9B5D-0FF89EF576FF

859795

Figs 3, 4, 5

##### Etymology.

The specific epithet ‘*khalidii*’ (Latin) is an honorific for “Prof. Dr. Abdul Nasir Khalid,” a pioneering mycologist from Pakistan whose contributions have been foundational to the study of macrofungal biodiversity in the region.

##### Holotype.

Pakistan • Khyber Pakhtunkhwa, Swat District, Aligrama Village, Tutbanie Forest; 34°48'00"N, 72°19'00"E, +/- 6 km; 908 m a.s.l.; 30 July 2024; Wajid Ali; under *Quercus leucotrichophora* Camus; GenBank accessions, ITS = PV608030, LSU = PV608041, tef1 = PV865529; AS-27, LAH38693.

**Figure 3.**
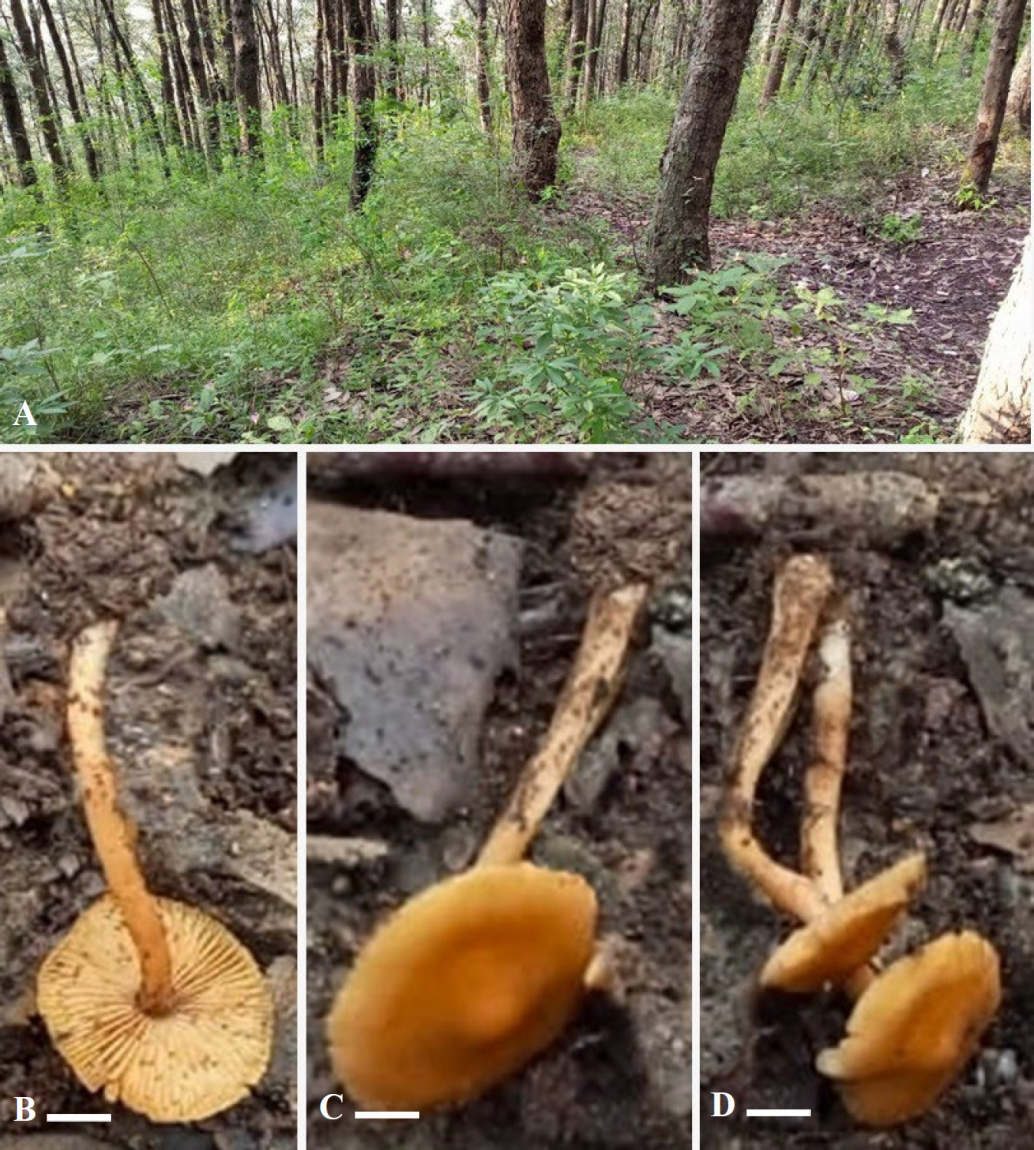
*Inocybe khalidii*. **A**. Habitat; **B–D**. Basidiomata. Scale bars: 7 μm (**B–D**).

##### Diagnosis.

*Inocybe khalidii* differs from phylogenetically and morphologically related species in having the subglabrous and vivid orange-brown pileus; the non-pruinose, slightly non-marginate bulbous stipe; and the rather large, broadly ellipsoid, and smooth basidiospores. This combination of morphological characters, together with the molecular profile of ITS, LSU, and *tef*-1 sequences, separates *Inocybe khalidii* from any known described species.

**Figure 4.**
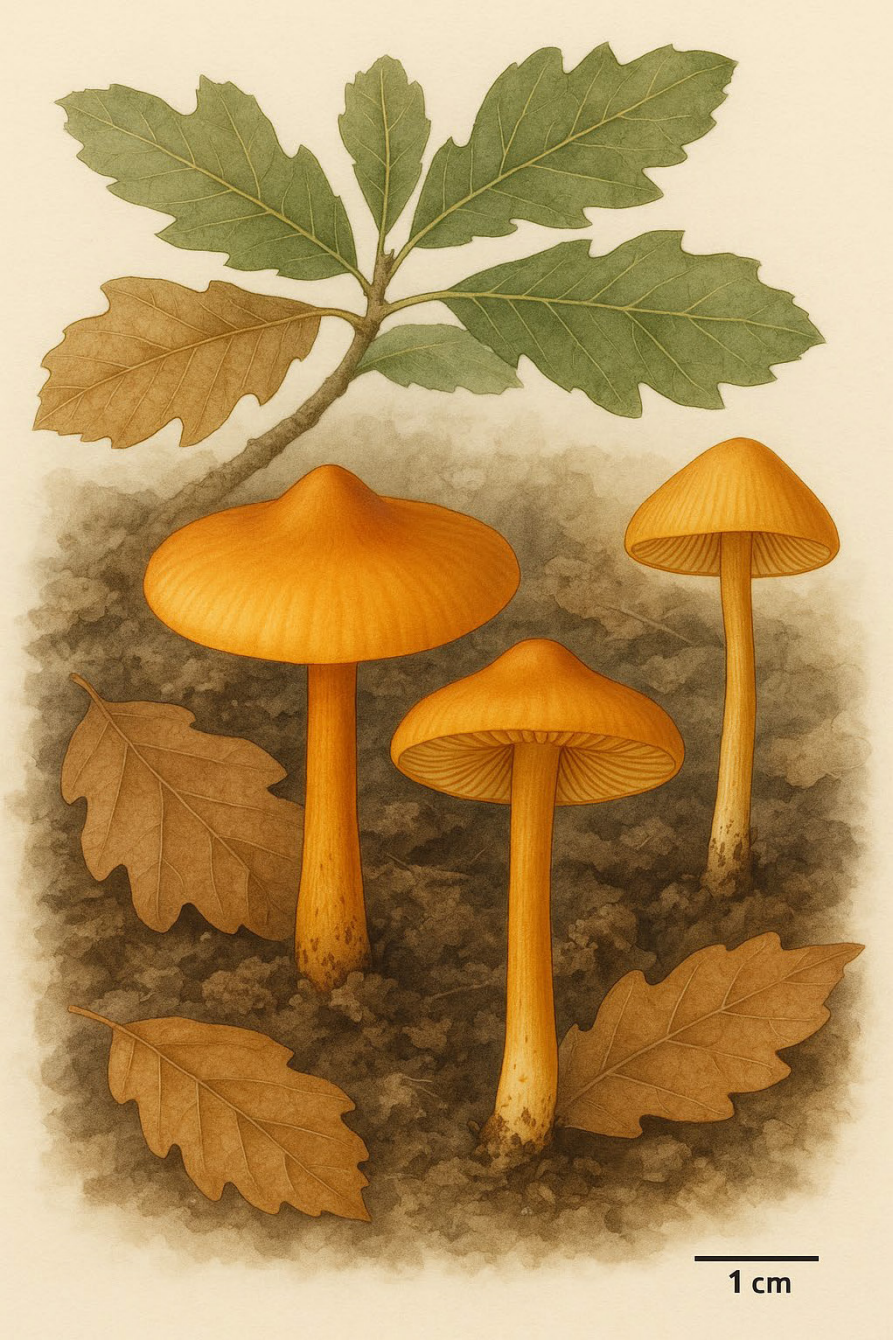
Illustration of *Inocybe khalidii*.

##### Description.

***Basidiomata*** collybioid. ***Pileus*** 5–16 mm diam., initially hemispherical, becoming planoconvex upon maturity with a prominent umbo, subglabrous to faintly fibrillose, vivid orange-brown (5YR 7/10 to 5YR 7/12), becoming light orange-brown (5YR 8/6) towards smooth to slightly rimose margins. ***Lamellae*** adnexed with entire edges, subdistant to close, light warm yellow (7.5YR 8/6). ***Lamellulae*** in a single tier. ***Stipe*** 10–20 × 2–4 mm, cylindrical, sometimes slightly decurved at the middle, subglabrous, vivid orange-brown (5YR 7/10) becoming light orange-brown (5YR 8/6) towards the slightly bulbous but non-marginated base and poorly pruinose apex. ***Context*** very light orange (7.5 YR 8/6).

**Figure 5.**
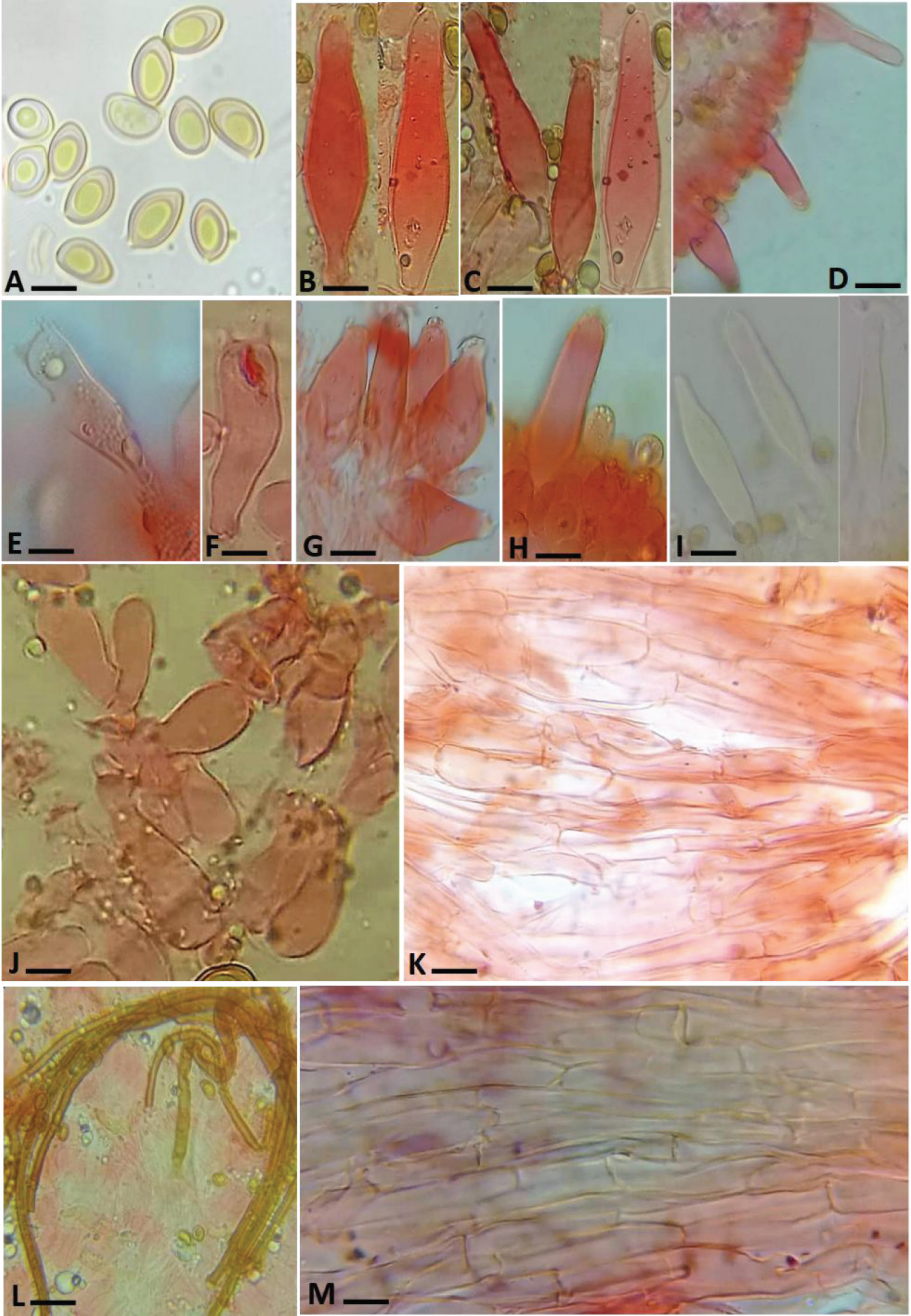
Anatomy of *Inocybe khalidii*. **A**. Basidiospores; **B–D**. Pleurocystidia; **E, F**. Basidia; **G, H**. Cheilocystidia; **I**. Pleurocystidia in KOH; **J**. Paracystidia; **K**. Pileipellis; **L**. Oleiferous hyphae; **M**. Stipitipellis. Scale bars: 10 μm (**A**); 7 μm (**B–D, I**); 6 μm (**E, F**); 7.5 μm (**G, H**); 15 μm (**K, M**); 18 μm (**L**).

***Basidiospores*** [40/2/2], (7.4–)8.9–10.9(14.3) × (6.2–)7.4–9.0(–10.3) μm, [avL × avW 10.86 × 8.24 μm, Q = 1.19–1.48, Q_av_ = 1.32], smooth, broadly ellipsoid to ellipsoid, sometimes subamygdaloid, mostly with obtuse apex, inamyloid. ***Basidia*** (excluding sterigmata) 21–34 × 6–9 μm, narrowly clavate to clavate, 2–4 spored. ***Pleurocystidia*** 37–64 × 11–24 μm, metuloid, wall 2.0–4.0 μm thick, narrowly utriform, narrowly lageniform, thick-walled, apex poorly crystalliferous or easily losing crystals, only faintly reacting in KOH. ***Cheilocystidia*** 32–44 × 9–33 μm, as above but more utriform-pyriform. ***Paracystidia*** 11–16 × 7–11 μm, narrowly clavate to broadly clavate. ***Pileipellis*** a cutis, hyphae 6–10 μm diam., septate. ***Stipitipellis*** a cutis, hyphae measuring 7–10 μm diam, septate, branched. ***Caulocystidia*** rare and present only at the extreme apex as a continuation of the lamellae edge. ***Oleiferous hyphae*** (5–7 μm, diam.) present in all structures. ***Clamp connections*** present and easily observed.

##### Habit and habitat.

Solitary on loamy soil (pH 6.3–6.5) under *Quercus leucotrichophora* Camus, in mountainous forests of mixed deciduous trees dominated by *Quercus* spp. and a few *Pinus* species.

##### Geographical distribution.

So far, it is only known from the Tutbanie and Zara Ghat forests, in the vicinity of Aligrama village, Swat District, Khyber Pakhtunkhwa Province, Pakistan.

##### Additional material examined.

Pakistan • Khyber Pakhtunkhwa, Swat District, Aligrama Village, ZaraGhat Forest; 34°48'00"N, 72°19'00"E, +/- 6 km; 908 m a.s.l.; 13 August 2023; Wajid Ali; under *Quercus leucotrichophora* Camus; GenBank accession, ITS = PV611493, LSU = PV639641; A-12, LAH38694.

#### Inocybe floribundae

Taxon classificationFungiAgaricalesInocybaceae

Farzana, Sana & Kiran sp. nov.

4181896D-EB63-5FBB-8598-78AAE7FB7C7B

861296

Figs 6, 7, 8

##### Etymology.

The specific epithet ‘*floribundae*’ (Latin) refers to the association of this species with *Quercus floribunda*.

##### Holotype.

Pakistan • Khyber Pakhtunkhwa, Swat District, Shawar Valley, Gat Shawar; 35°26'22"N, 72°28'38"E, +/- 6 km; 2100 m a.s.l.; 07 August 2023; Wajid Ali and Munazza Kiran; under *Quercus floribunda* Lindl. ex A.; GenBank accessions, ITS = PX630063, LSU = PX630065, GS-11, LAH38691.

##### Diagnosis.

*Inocybe floribundae* is distinguished from its phylogenetic allies by the beige to brown, distinctly scaly pileus, bulbous but weakly-marginate stipe, apically restricted caulocystidia, utriform-obclavate cystidia, and subisodiametric, bluntly nodulose basidiospores. Moreover, it is geographically separated from other species in the Xanthomelas group that occur in Europe, North America, and Australia, except for *I. populea* in East Asia (Japan), whereas the new species is endemic to South Asia (Pakistan).

**Figure 6.**
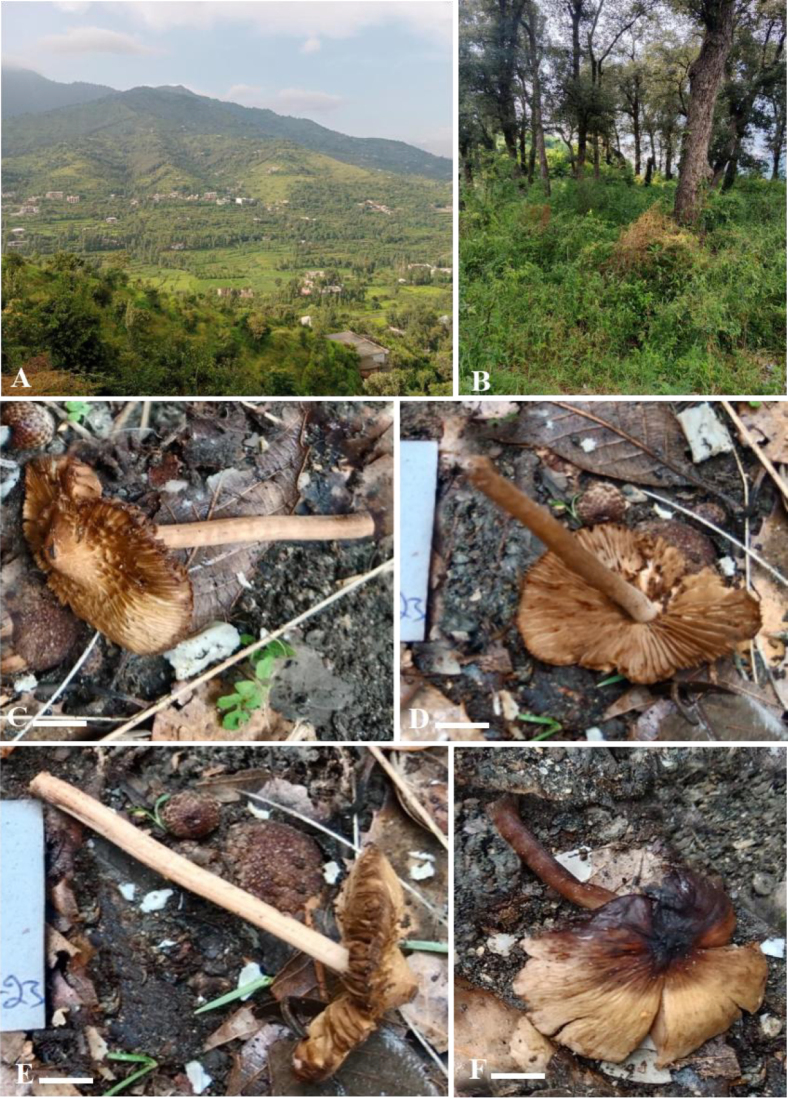
**A, B**. Sampling site; **C–E**. Basidiomata of *Inocybe floribundae* holotype; **F**. Basidiomata of *Inocybe floribundae* paratype. Scale bars: 14 μm (**C–F**).

##### Description.

***Basidiomata*** medium to large-sized. ***Pileus*** 64–94 mm diam., subconical-campanulate to applanate or slightly plano-convex with papillate center in maturity. Papillae more obvious when mature than in the early stages, subglabrous when young, becoming clearly fibrillose to squamulose at maturity, especially around the papilla, beige to light brown (7.5YR 6/6), margins rimose and striate in maturity. ***Lamellae*** slightly adnate with entire edges, sub-distant to close, light brown (7.5YR 6/6). ***Lamellulae*** absent. ***Stipe*** 32–69 × 4–7 mm, cylindrical with a barely or non-marginated bulbous base, smooth to very slightly fibrillose, pruinose mostly at apex, beige to orange brown (7.5YR 8/6) to moderate brown (7.5YR 5/6). ***Context*** light yellowish brown.

**Figure 7.**
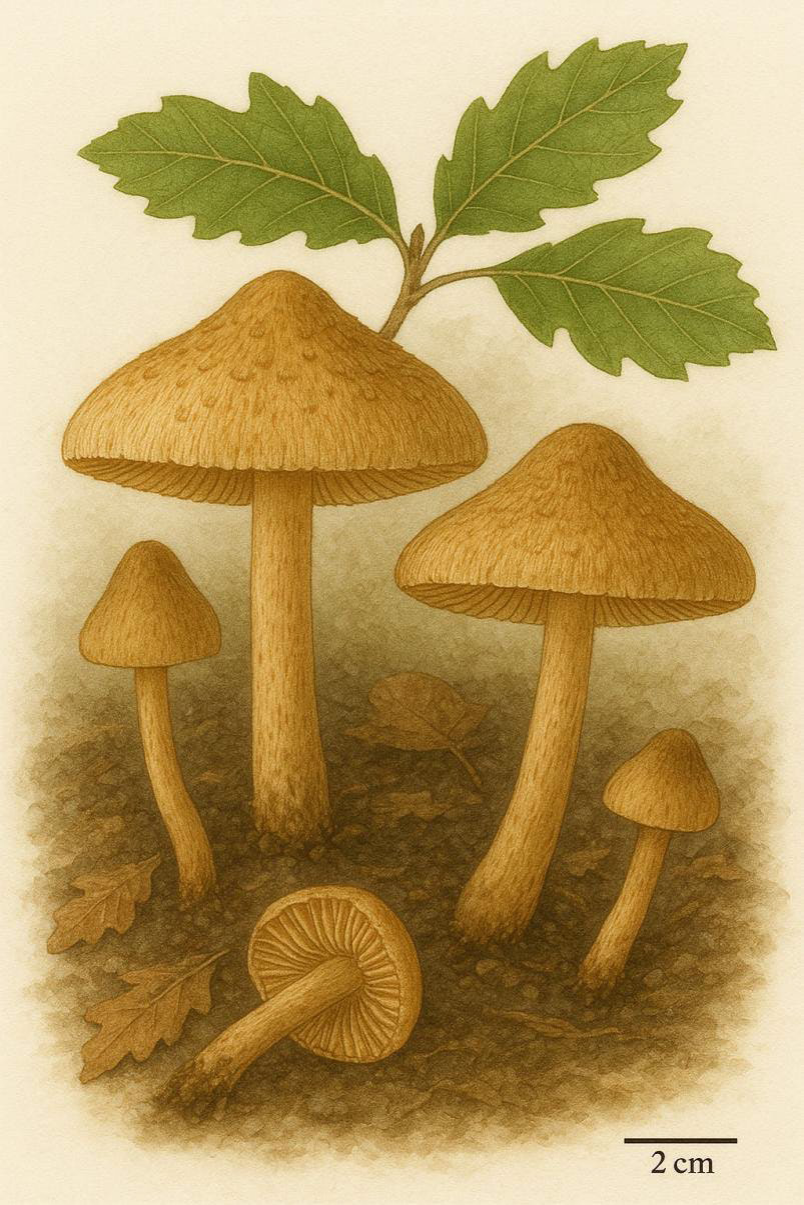
Illustration of *Inocybe floribundae*.

***Basidiospores*** [40/2/2], (7.3–)8.8–9.6(–12.2) × (5.4–)6.1–7.2(–8.9) μm, avL × avW 10.25 × 7.66 μm, Q = 1.34–1.41, Q av = 1.36, nodulose, knobs blunt, a few sharp, 0.7–1.1 μm high, hexadiametric, inamyloid. ***Basidia*** (excluding sterigmata) 32–49 × 9–12 μm, narrowly clavate to clavate, 2–4 spored. ***Pleurocystidia*** 43–67 × 19–29 μm, metuloid, obclavate (inversely clavate), thick-walled bearing crystalliferous apex, hyaline in KOH. ***Cheilocystidia*** 31–52 × 9–18 μm, narrowly lageniform, a few narrowly obclavate (inversely narrowly clavate) with crystalliferous apex. ***Paracystidia*** 14–20 × 8–13 μm, narrowly clavate to broadly clavate. ***Pileipellis*** a cutis, hyphae 12–14 μm diam., septate, unbranched. ***Stipitipellis*** a cutis, hyphae measuring 6–9 μm diam, septate, unbranched. ***Caulocystidia*** present only at apex, abundant, fusiform to ventricose, a few obclavate. ***Oleiferous hyphae*** (4–6 μm, diam.), abundant in pileipellis and stipitipellis. ***Clamp connection*s** abundant.

**Figure 8.**
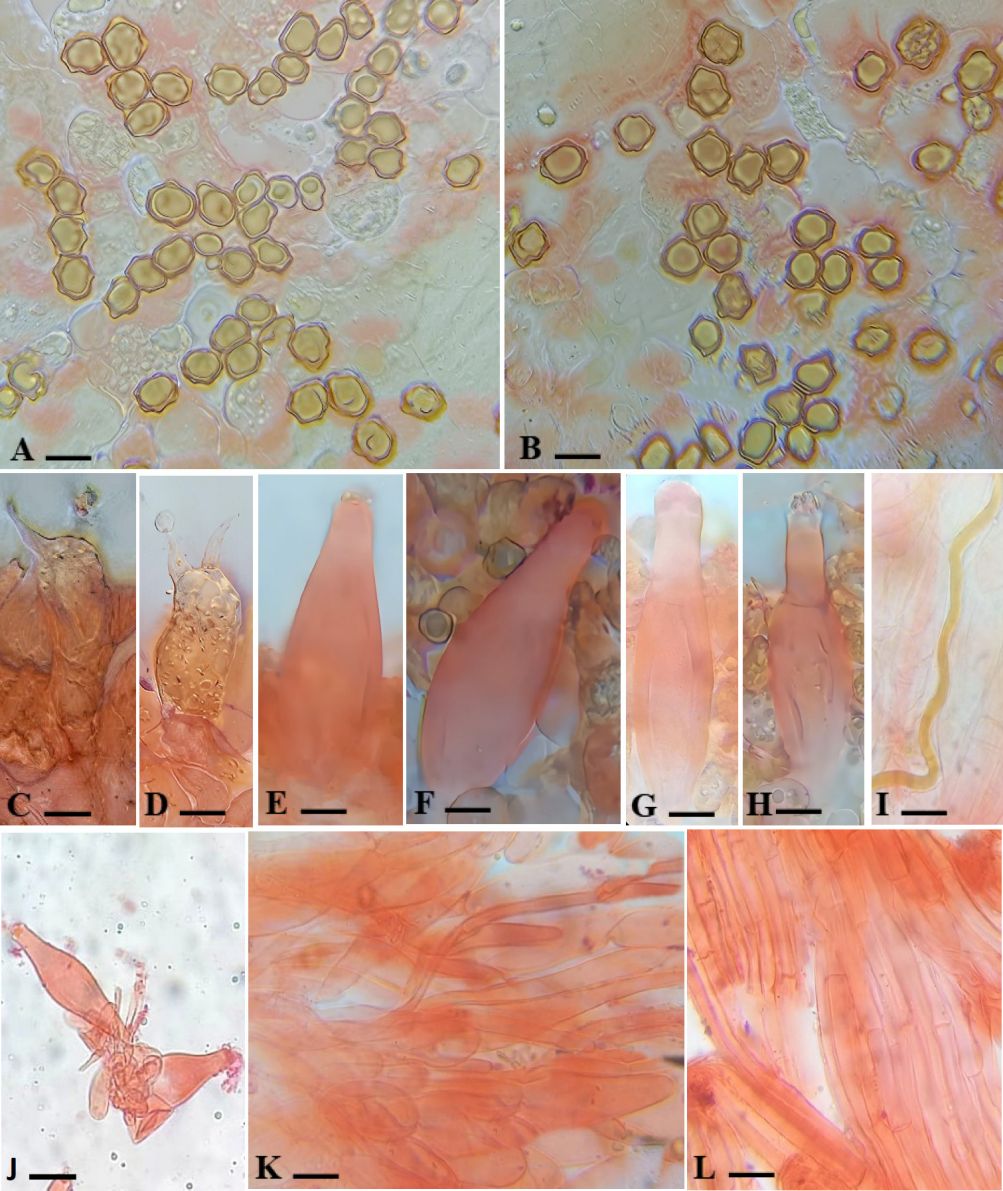
Anatomy of *Inocybe floribundae*. **A, B**. Basidiospores; **C, D**. Basidia; **E, F**. Pleurocystidia; **G, H**. Cheilocystidia; **I**. Oleiferous hyphae; **J**. Caulocystidia; **K**. Pileipellis; **L**. Stipitipellis. Scale bars: 12 μm (**A, B**); 8 μm (**C, D**); 7 μm (**E, F**); 9 μm (**G, H**); 19 μm (**I, L**); 11 μm (**J**); 14 μm (**K**).

##### Habit and habitat.

Solitary on loamy (pH 6.4–6.7) soil under *Q. floribunda* in a forest dominated by *Quercus* spp. and a few *Pinus* species.

##### Geographical distribution.

So far, it is only known from the Shawar Valley and Aligrama Village, Swat District, Khyber Pakhtunkhwa Province, Pakistan.

##### Additional material examined.

Pakistan • Khyber Pakhtunkhwa, Swat District, Aligrama Village; 34°48'00"N, 72°19'00"E, +/- 6 km; 908 m a.s.l.; 13 July 2023; Wajid Ali; under *Quercus floribunda*; GenBank accessions, ITS = PX630064, LSU = PX630062; AS-32, LAH38692.

### Checklist of *Inocybaceae* of Pakistan

This checklist is organized into two distinct sections based on the method of description. Section 1 comprises species of *Inocybaceae* described or recorded from Pakistan based on morpho-anatomical and phylogenetic evidence, while Section 2 includes species identified solely based on macromorphology from the country (Table 2).

**Table 2.** Checklist of *Inocybaceae* reported and recorded from Pakistan.

Section 1 (*Inocybaceae* species reported from Pakistan based on morpho-anatomy and phylogenetic evidence - 37 species)							
Taxon name	Locality	Habit and habitat	Collection date	GenBank no.	Voucher no.	MycoBank no.	Reference
**Genus *Inocybe*** (15 *Inocybe* species have been reported from Pakistan to date based on morpho-anatomy and phylogeny)							
*Inocybe alboaurantiaca* Sana & M. Kiran, in Sana, Kiran, Okabe, Hafazallah, Javeed & Ali, Nordic J. Bot. 2025(e04914): 3 (2025)	Aligrama, Swat District, KP Province	Solitary on ground in *Quercus* dominated forest	20 July 2023, 31 July 2023	PQ805433 PQ858028 PV489868 PV489871	LAH38556 LAH38557	MB875331	Sana et al. (2025a)
*Inocybe ahmadii* Farooqi, Niazi & Khalid, in Farooqi, Aqdus, Niazi, Jabeen & Khalid, Mycotaxon 132(2): 259 (2017)	Khanspur, Abbottabad District, KP Province	Solitary on ground under Pinus wallichiana A.B. Jacks.	14 Aug 2015	KX254462	LAH14215	MB817163	Farooqi et al. (2017)
*Inocybe bhurbanensis* Naseer, Razzaq, Saeed and Khalid in Naseer, Razzaq, Saeed & Khalid, Nordic J. Bot. 2023(e04045): 9 (2023)	Bhurban Town and Patriata, Rawalpindi District, Punjab Province.	Solitarily under pine trees	04 Sep 2020, 07 Sep 2020, 19 Sep 2022	OQ152810 OQ152811 OQ152812 OQ152943 OQ152944	LAH0000	MB846875	Naseer et al. (2023b)
*Inocybe caroticolor* T. Bau & Y.G. Fan, Mycotaxon 123: 170 (2013)	Shawar valley, Swat District, KP Province	On soil under *Quercus dilatata* Lindl. ex Royle and *Q. oblongata* D.Don	14 July 2014, 25 Aug 2014, 15 Aug 2015	MH473144 MH473145 MH473146 MH473148 MH536983 MH536984 MH536985 MH536986	LAH35266 LAH35268 LAH35269	-	Naseer et al. (2019)
*Inocybe crenata* U. Ashraf, Afshan, A. Razzaq, M. Ali, Niazi, Naseer & Khalid, Mycobiology: 10.1080/12298093.2024.2398273, 3 (2025)	Ayubia National Park, Abbottabad District, KP Governor house, Bhurban town, Rawalpindi District, Punjab Province	Solitary on organic matter rich soil near *Abies pindrow* (Royle ex D.Don) Royle, *Cedrus deodara* (Roxb. ex D.Don) G.Don, *P. roxburghii* Sarge. and *Q. incana* Roxb.	14 July 2019, 28 July 2019, 07 Sep 2020, 30 July 2021	OQ826579 OQ826580 OQ826582 OQ826583 OR543352 OR534594 OR534595	PUL00044680 LAH37821 LAH37822 LAH35292	MB850056	Ashraf et al. (2025)
*Inocybe cryptocystis* D.E. Stuntz, Pap. Mich. Acad. Sci. 39: 58 (1954) [1953]	-	Associated with *P. wallichiana*	-	KF679812	-	-	Hanif et al. (2022)
*Inocybe khanspurensis* Naseer, Brearley, Faryad & Khalid, in Naseer, Jabeen, Brearley, Faryad, Asghar & Khalid, Plant Biosyst. 160:94 (2026)	Khanspur, Abbotabad District, KP Province	-	21 July 2023, 16 Aug 2024	PQ826296 PQ814310 PQ879974 PQ814311	LAH38552 LAH38510	MB857042	Naseer et al. (2026)
*Inocybe kohistanensis* Jabeen, I. Ahmad & Khalid, in Jabeen, Ahmad, Rashid & Khalid, Turk. J. Bot. 40: 313 (2016)	Kalam and Mashkun, Swat District, KP Province	Under *C. deodara*	4 Sep 2013, 5 Sep 2013	KP316243 KP316244 KP316245 KT897911	LAH35001 LAH35002 LAH35003 LAH35024	MB812275	Jabeen et al. (2016)
*Inocybe leptocystis* G.F. Atk., Am. J. Bot. 5: 212 (1918)	Miandam, Swat District, KP Province, Pakistan	Solitary under conifers	23 Aug 2015	KX254461	LAH35112	-	Farooqi et al. (2017)
*Inocybe napipes* J.E. Lange, Dansk bot. Ark. 2(no. 7): 44 (1917)	Rama forest, Astore District, Gilgit Baltistan Province, Sharan forest, Kaghan Valley and Kamalban, Mansehra District, KP Province	Solitary or in small group on soil	24 Aug 1989, 24 Sep 1990	-	PMNH 8422 PMNH 8249	-	Sultana et al. (2011); Razaq and Shehzad 2017)
*Inocybe nigrosquamulosa* Naseer, Brearley, Faryad & Khalid in Naseer, Jabeen, Brearley, Faryad, Asghar & Khalid, Plant Biosyst. 160:94 (2026)	Khanspur, Abbotabad District, KP Province	-	21 July 2021, 21 July 2023	PQ826299 PQ879975 PQ814320 PQ814348	LAH38553 LAH 38511	MB857302	Naseer et al. (2026)
*Inocybe nitidiuscula* (Britzelm.) Lapl., Dict. iconogr. champ. sup. (Paris): 523 (1894)	Koza Gali, Ayubia, Galyat, KP Province	Gregarious, on moist ground under *Alnus nitida* (Spach) Endl.	23 Aug 2010	HE862959	LAH230817	-	Ilyas et al. (2013)
*Inocybe parva* Naseer, Asghar & Khalid in Naseer, Jabeen, Brearley, Faryad, Asghar & Khalid, Plant Biosyst. 160:94 (2026)	Khanspur, Abbotabad District, KP Province	-	23 Aug 2022,	PQ826303 PQ885559 PQ826305 PQ859504	LAH38509 LAH381710	MB857311	Naseer et al. (2026)
Section 1 (*Inocybaceae* species reported from Pakistan based on morpho-anatomy and phylogenetic evidence - 37 species)							
Taxon name	Locality	Habit and habitat	Collection date	GenBank no.	Voucher no.	MycoBank no.	Reference
*Inocybe subhimalayana* Razzaq, Naseer & Khalid [as ‘subhimalayanensis’], European Journal of Taxonomy 870: 78 (2023)	Bhurban town, Rawalpindi District, Punjab Province Murree, Murree District, Punjab Province	On soil below *Pinus*	18 Sep 2019, 07 Sep 2020, 12 Sep 2020, 04 Sep 2021, 10 Sep 2021	ON810645 ON810644 ON810645 ON810646 ON810647 ON810648 ON810652 ON810653 ON911331	LAH37437 LAH37438 LAH37439 LAH37440 LAH15167	MB845094	Razzaq et al. (2023)
*Inocybe swatensis* I. Ahmad & A.N. Mill. In Ahmad, Miller, Khan, Khan & Khalid, Pak. J. Bot. 57: 6 (2025)	Nalkoy region, Matta Tehsil, Swat District, KP Province	On moss covered soil of mixed coniferous forests dominated by *P. wallichiana* and *A. pindrow*.	08 Aug 2019, 09 Aug 2019	OR625717 OR625718 OR625719 OR625720	ICFP # SK1900 ICFP # SK1901	MB851471	Ahmad et al. (2025)
**Genus *Inosperma*** (3 *Inosperma* species have been reported from Pakistan to date based on morphoanatomy and phylogeny)							
*Inosperma kashmiranum* Naseer, Khurshid & Khalid, in Naseer, Khurshid, Fan & Khalid, BMC Microbiology 25:688 (2025)	Nari, Bagh District, Azad Jammu and Kashmir	Solitary on soil in mixed forest of *P. wallichiana*, *C. deodara*, *Quercus* spp. and *A. pindrow*	29 Aug 2021, 01 Sep 2022	PQ449774 PQ449776 PQ452890 PQ584605	LAH38340	MB856183	Naseer et al. (2025)
*Inosperma pakistanicum* Sana & M. Kiran, in Sana, Kiran & Ali, Phytotaxa 690(1): 82 (2025)	Aligrama village, Swat District, KP Province	On soil under *Quercus* spp.	30 July 2023, 01 Aug 2023	PQ452107 PQ452108 PQ614170	LAH38334 LAH38335	MB856210	Sana et al. (2025b)
*Inosperma shawarense* (Naseer & Khalid) Aïgnon & Naseer, in Aïgnon, Jabeen, Naseer, Yorou & Ryberg, MycoKeys 77: 111 (2021)	Shawar valley, Swat District, KP Province	Solitary on ground under *Q. oblongata*	14 July 2015	KY616964 KY616965 KY616966	FLAS-F-S9456 LAH35195	MB820130	Aïgnon et al. (2021)
**Genus *Mallocybe*** (8 *Mallocybe* species have been reported from Pakistan to date based on morphoanatomy and phylogeny)							
*Mallocybe ahmadii* I. Rauf & Saba, in Crous et al., Persoonia 50: 259 (2023)	Quaid-e-Azam University Botanical Garden, Islamabad	Under *P. roxburghii*	05 Aug 2021, 08 Dec 2022	OP997541 OP997542 OP997544 OP997545	LAH37801 LAH37802	MB848105	Crous et al. (2023)
*Mallocybe delecta* (P. Karst.) Matheny & Esteve-Rav., in Matheny, Hobbs & Esteve-Raventós, Mycologia 112(1): 106 (2019)	Bargai, Kumrat Valley, Upper Dir District, KP Province	On forest floor	22 Aug 2017	PV652479 PV652480	LAH35925	-	Kiran et al. (2025)
*Mallocybe himalayana* Y.G. Fan, Khurshid & Naseer, in Naseer, Khurshid, Fan & Khalid, Phytotaxa 640(3): 257 (2024)	Nari, Bagh, Saliyan, Azad Jammu and Kashmir	On soil	29 Aug 2021, 01 Sep 2021	OQ448897 OQ448900 OQ448901 OQ448902	LAH37696 LAH37697	MB847642	Naseer et al. (2024)
*Mallocybe kashmirana* Khurshid, Naseer & Khalid, in Naseer, Khurshid, Fan & Khalid, Phytotaxa 640(3): 260 (2024)	Nari, Bagh, Saliyan, Azad Jammu and Kashmir	-	29 Aug 2021	OQ458716 OQ448896 OQ448898	LAH37695	MB847643	Naseer et al. (2024)
*Mallocybe luteoaurantiaca* Sattar, M. Kiran & Khalid, in Kiran, Sana, Sattar & Khalid, Phytotaxa 720(1): 9 (2025)	Bargai, Kumrat Valley, Upper Dir District, KP Province	On forest floor	22 Aug 2017	PV652465 PV652467	LAH35911	MB859265	Kiran et al. (2025)
*Mallocybe pakistanica* Saba & Khalid, in Saba, Khalid & Sarwar, MycoKeys 99: 177 (2023)	Chattar Plain, Mansehra District, KP Province	Under *P. wallichiana*	22 Sep 2013, 02 Sep 2015	OK360951 OK360952 OK360953 OQ458716 OK392118 OK392119 OK392120	ISL-F002 ISL-F003 ISL-F004	MB843490	Saba et al. (2023)
*Mallocybe pinicola* Saba & Khalid, in Saba, Khalid & Sarwar, MycoKeys 99: 179 (2023)	Chattar Plain, Mansehra District, KP Province	Under *P. wallichiana*	22 Sep 2013, 02 Sep 2015	OK360954 OK360955 OK360956 OK392121 OK392122 OK392123	ISL-F005 ISL-F006 ISL-F007	MB843491	Saba et al. (2023)
*Mallocybe velutina* Saba & Khalid, Mycoscience 61(6): 350 (2020)	Thandiani, Abbottabad District, KP Province	In groups, scattered under *P. wallichiana*	15 Sep 2012, 22 Sep 2013, 13 Sep 2017	MK990129 MK990130 MK990131 MK999927 MK999928 MK999929	LAH310057 LAH310058 LAH310060	MB823035	Saba and Khalid (2020)
Section 1 (*Inocybaceae* species reported from Pakistan based on morpho-anatomy and phylogenetic evidence - 37 species)							
Taxon name	Locality	Habit and habitat	Collection date	GenBank no.	Voucher no.	MycoBank no.	Reference
**Genus *Pseudosperma*** (11 *Pseudosperma* species have been reported from Pakistan to date based on morphoanatomy and phylogeny)							
*Pseudosperma albobrunneum* Jabeen, Zainab, H. Bashir & Khalid, Mycotaxon 136(2): 364 (2021)	Darosh, Lower Dair District, KP Province; Kalam and Mashkun, Swat District, KP Province Khanian, Mansehra District, KP Province	On soil under *C. deodara* and *P. wallichiana*	04 Sep 2013, 05 Sep 2013, 05 Aug 2014, 04 Sep 2015	MG495392 MG495393 MG495395 MG495394 MG495396	LAH35045 LAH35046 LAH35047 LAH35288 LAH35289	MB840056	Jabeen et al. (2021)
*Pseudosperma aureocitrinum* (Esteve-Rav.) Matheny & Esteve-Rav., in Matheny, Hobbs & Esteve-Raventós, Mycologia 112(1): 109 (2019)	Patriata, Rawalpindi District, Punjab Province	Gregarious under *P. wallichiana*	04 Sep 2020	PQ721832	LAH38506	-	Faryad et al. (2025)
*Pseudosperma brunneoumbonatum* Saba & Khalid, in Saba, Haelewaters, Pfister & Khalid, MycoKeys 69: 11 (2020)	Shimla, Abbottabad District, KP Province	Solitary or in groups, scattered on the forest floor in stands of *P. roxburghii*	Aug and Sep	MG742419 MG742420 MG742421	LAH310032	MB822655	Saba et al. (2020)
*Pseudosperma flavorimosum* Jabeen & Khalid, Mycotaxon 135(1): 187 (2020)	Kaghan valley, Mansehra District, KP Province Mashkun, Swat District, KP Province	On soil under *P. wallichiana* and *C. deodara*	05 Sep 2013, 03 Aug 2014	MG495391	LAH35042 LAH35043	MB823494	Jabeen et al. (2020)
*Pseudosperma himalayense* (Razaq, Khalid & Takah. Kobay.) Matheny & Esteve-Rav., in Matheny, Hobbs & Esteve-Raventós, Mycologia 112(1): 111 (2019)	Khanspur, Abbottabad District, KP Province Fairy Meadows forest, Chilas District, Gilgit Baltistan Province	On soil under *P. wallichiana*	22 July 2010, 23 Aug 2010, 30 Aug 2010	MH745138	LAH210710 LAH230810	MB827821	Liu et al. (2018)
*Pseudosperma khanspuricum* Naseer, Faryad & Khalid in Faryad, Asghar, Razzaq, Ayub, Naseer & Khalid, Nordic J. Bot. 2025(e04757): 2 (2025)	Khanspur, Abbottabad District, KP Province	In mixed coniferous forest	21 July 2023, 23 July 2023	PQ637574 PQ654718 PQ654720 PQ721844	LAH38503 LAH38504 LAH38505	MB856969	Faryad et al. (2025)
*Pseudosperma pakistanense* (Z. Ullah, Jabeen, H. Ahmad & Khalid) Matheny & Esteve-Rav. in Matheny, Hobbs & Esteve-Raventós, Mycologia 112(1): 113 (2019)	Lower Shawar, Swat District, KP Province	On soil under *Q. incana*	28 April 2016 28 July 2016	MF575848 MF575849 MF588965 MG958608 MG958609	LAH35283 LAH35284 LAH35285	MB822128	Ullah et al. (2018)
*Pseudosperma pinophilum* Saba & Khalid in Saba, Haelewaters, Pfister & Khalid, MycoKeys 69: 18 (2020)	Shimla, Abbottabad District, KP Province, Yakh Tangay Shangla District, KP Province	On soil under *P. wallichiana*	14 Sep 2012, 02 Sep 2013	MG742414 MG742415 MG742416 MG742417 MG742418 MK474612	FH 00304582 LAH 310049	MB822656	Saba et al. (2020)
*Pseudosperma quercinum* Naseer & Jabeen in Naseer, Jabeen, Ashfaq, Akbar, Hussain & Khalid, Phytotaxa 622(4): 262 (2023)	Malam Jabba valley, Swat District, KP Province Parachinar, Kurram District, KP Province Malam Jabba valley, Swat District, KP Province	Solitary on rich loamy soil under *Quercus* spp.	10 Aug 2016, 31 July 2018, 22 Aug 2019	OP303370 MZ314058 MZ314059 MZ314078 MZ314079	LAH35232 LAH35233 LAH37418	MB849661	Naseer et al. (2023a)
*Pseudosperma triaciculare* Saba & Khalid, in Saba, Haelewaters, Pfister & Khalid, MycoKeys 69: 20 (2020)	Batrasi, Mansehra District, KP Province Shimla, Abbottabad District, KP Province	Solitary or in groups, scattered on the forest floor in stands of *P. roxburghii*	14 Sep 2012, 03 Aug 2014	MG742423 MG742424 MG742425 MG742426 MG742427 MG742428 MG742429 MG742430 MG742431	FH 00304561 LAH31004 LAH31005 LAH31006	MB822657	Saba et al. (2022)
*Pseudosperma umbonatum* Jabeen, Razzaq, Aqdas, Naseer & Khalid, in Jabeen, Razzaq, Aqdas, Iqbal, Ijaz, Izhar, Kamran, Naseer & Khalid, Phytotaxa 701(1): 30 (2025)	Murree hills, Murree District, Rawalpindi division, Punjab	Solitary or scattered, in mixed pine forests (*Pinus roxburghii*, *P. wallichiana*, with deciduous trees like *Q. incana* and *Q. dilatata*) on nutrient-rich soil	12 August 2018	PP526036 PP526037	LAH36881	MB852970	Jabeen et al. (2025)
Section 2 (*Inocybaceae* species reported from Pakistan based on macro-morphology -18 species)							
Taxon Name	Locality	Habit and habitat		Collection date	Voucher No	Reference	
**Genus *Inocybe*** (13 *Inocybe* species have been reported from Pakistan to date based on morphology)							
*Inocybe argillacea* (Pers.) Singer, Persoonia 2(1): 8 (1961)	-	Under *Terminalia arjuna* (Roxb. ex DC.) Wight & Arn.		-	-	Ahmad et al. (1997)	
*Inocybe asterospora* Quél., Bull. Soc. Bot. France 26: 50 (1880) [1879]	Naran and Shogran, Mansehra District, KP Province	On the ground in pine forest		25 Aug 1989	PMNH8030	(Ahmad et al. 1997; Sultana et al. 2011)	
*Inocybe fibrosa* (Sowerby) Gillet, Hyménomycètes (Alençon): 517 (1876) [1878]	Naran, Mansehra District, KP Province	On soil		10 July 1989	PMNH 8168	Sultana et al. (2011)	
*Inocybe flocculosa* Sacc., Syll. Fung. (Abellini) 5: 768 (1887)	-	-		-	-	Ahmad et al. (1997)	
*Inocybe fuscidula* Velen., České Houby (Praze) 2: 378 (1920)	Naran, Mansehra District, KP Province	On humus		10 July 1989	-	Sultana et al. (2011)	
*Inocybe geophylla* P. Kumm. [as ‘*geophyllus*’], Führ. Pilzk. (Zerbst): 78 (1871)	Azad Kashmir and Naran, Mansehra District, KP Province Hunza District Gilgit Baltistan Province	On the ground under *Quercus* spp. Trooping, on soil among grasses		25 Aug 1989	PMNH 8242	(Ahmad et al. 1997; Sultana et al. 2011; Razaq and Shehzad 2017)	
*Inocybe glabripes* Ricken., Die Blätterpilze: 107 (1911)	Naran, Mansehra District, KP Province	On soil		25 Aug 1989	PMNH 8039	Sultana et al. (2011)	
*Inocybe hirtella* Bres., Fung. trident. 1(4-5): 52 (1884)	Sharan forest, Kaghan Valley, Mansehra District, KP Province	On soil		24 Aug 1989	PMNH 8031	Sultana et al. (2011)	
*Inocybe oblectabilis* (Britzelm.) Sacc., Syll. Fung. (Abellini) 11: 54 (1895)	-	-		-	-	Ahmad et al. (1997)	
*Inocybe posterula* (Britzelm.) Sacc., Syll. Fung. (Abellini) 5: 778 (1887)	Khanspur, Abbottabad District, KP Province	On the ground		-	-	Ahmad et al. (1997)	
*Inocybe praetervisa* Quél., in Bresadola, Fung. trident. 1(3): 35 (1883)	Lalazar, Mansehra District, KP Province	On soil		27 Sep 1990	PMNH 8396	Sultana et al. (2011)	
*Inocybe pyriodora* (Pers.) P. Kumm., Führ. Pilzk. (Zerbst): 79 (1871)	Murree, Murree District, Punjab Province Shogran and Mansehra District, Khyber Pakhtunkhwa Province	On the ground in coniferous forest (*Cedrus*-*Pinus*-*Abies*-*Picea*) under *P. excelsa* Wall. ex Royle		-	-	Ahmad et al. (1997)	
*Inocybe vaccina* Kühner., Führ. Pilzk. (Zerbst): 79 (1871)	Lalazar, Mansehra District, KP Province; Upper Bela, Lasbela District, Balochistan Province	On humus and soil		24 Aug 1889, 25 Sep 1990	PMNH 8036 PMNH 8460	Sultana et al. (2011)	
**Genus *Inosperma*** (3 *Inosperma* species have been reported from Pakistan to date based on omorphology)							
*Inosperma adaequatum* (Britzelm.) Matheny & Esteve-Rav., in Matheny, Hobbs & Esteve-Raventós, Mycologia 112(1): 101 (2019)	Lalazar, Mansehra District, KP Province	On humus of pine needles		28 Aug 1989	PMNH 8038	Sultana et al. (2011)	
*Inosperma bongardii* (Weinm.) Matheny & Esteve-Rav., in Matheny, Hobbs & Esteve-Raventós, Mycologia 112(1): 101 (2019)	-	-		-	-	Ahmad et al. (1997)	
*Inosperma erubescens* (A. Blytt) Matheny & Esteve-Rav., in Matheny, Hobbs & Esteve-Raventós, Mycologia 112(1): 102 (2019)	Khanspur, Abbottabad District, KP Province	On the ground under *A. pindrow*		26 Sep 1990	PMNH 8531	(Ahmad et al. 1997; Sultana et al. 2011)	
**Genus *Mallocybe*** (2 *Mallocybe* species have been reported from Pakistan to date based on morphology)							
*Mallocybe agardhii* (N. Lund) Matheny & Esteve-Rav., in Matheny, Hobbs & Esteve-Raventós, Mycologia 112(1): 105 (2019)	Dichal nallah (Dashkin), District Astore, Gilgit Baltistan Province	-		-	Solitary or in small trooping groups on soil	Razaq and Shehzad (2017)	
*Mallocybe fibrillosa* (Peck) Matheny & Esteve-Rav., in Matheny, Hobbs & Esteve-Raventós, Mycologia 112(1): 106 (2019)	-	-		-	-	Ahmad et al. (1997)	

The family *Inocybaceae* comprises 55 accepted species in Pakistan. Within *Inocybaceae*, species from four genera have been documented. The genus *Inocybe* comprises 28 species, of which 15 have been identified using both morphology and phylogeny and 13 based solely on morphology. The genus *Inosperma* includes six species, three supported by morphological and phylogenetic analyses and three by morphology alone. *Mallocybe* comprises 10 species, eight of which have been confirmed using both morphological and molecular evidence, while two are based solely on morphology. *Pseudosperma* is represented by 11 species, all of which have been described using an integrative morpho-phylogenetic approach. No species have yet been reported from the remaining genera of *Inocybaceae*—*Auritella*, *Nothocybe*, and *Tubariomyces*—in Pakistan. Lastly, one name, *Inocybe inocybium*, was described by Ahmad et al. (1997); however, this name does not appear in Index Fungorum, MycoBank, or Fungal Names.

## Discussion

This study presents a comprehensive checklist of the family *Inocybaceae* from Pakistan. Previously, a varied number of species have been listed in the literature, but in this study, we tried to consult the available literature thoroughly and found 28, six, ten, and 11 valid species of the genera *Inocybe*, *Inosperma*, *Mallocybe*, and *Pseudosperma*, respectively, reported from Pakistan to date (data retrieved on 27 March 2026). Moreover, no *Auritella*, *Nothocybe*, or *Tubariomyces* species have been reported from the country to date. Although species of these genera have been reported from neighboring countries, including India, they may also be discovered in Pakistan. The occurrence of *Inocybaceae* in Pakistan is supported by the country’s diverse climatic zones and rich ectomycorrhizal forests dominated by *Pinus*, *Cedrus*, *Quercus*, *Abies*, and *Picea*. These broad altitudinal habitats provide ideal conditions for *Inocybaceae* to thrive (Jabeen et al. 2014; Khan 2019). Therefore, many more species are likely present but remain undocumented across various regions of Pakistan.

This research also explores a macrofungal hotspot (Swat), leading to the discovery of two new *Inocybe* species (*I. khalidii* and *I. floribundae*). In the ITS+LSU-based phylogram, *I. khalidii* appears as a strongly supported (PP = 1.00/BP = 99) sister taxon to a clade containing several allopatric species from Mexico, Japan, and the USA. This clade is nested within a broader lineage that includes other well-supported species such as *I. furfurea*, *I. rufescens*, *I. agroterae*, *I. catalaunica*, and *I. rivierana*. In the *tef*1-based phylogram, *I. khalidii* formed a moderately supported (PP = 1.00/BP = 50) sister clade to *I. subexilis* from the USA. The weak support for *Inocybe khalidii* in the *tef*1-based phylogeny may be attributed to the limited number of sequences available for this gene within the genus, resulting in insufficient taxon sampling and reduced phylogenetic resolution. *Inocybe floribundae* appears as a species-level clade, sister to *I. corsica* and *I. diabolica*, with maximum statistical support (PP = 1.00/BP = 100). Other phylogenetically allied species to *I. floribundae* are *I. flavobrunnescens*, *I. similis*, *I. obtusiuscula* Kühner, *I. bidumensis* E. Larss. & Vauras, *I. intricata* Peck, *I. subrimosa* (P. Karst.) Sacc., *I. strickeriana* Bandini, Anja Schneid. & M. Scholler, *I. salicis* Kühner, *I. populea* Takah. Kobay. & Courtec., and *I. lacunarum* Vauras & E. Larss.

*Inocybe rufescens* (NLB834, holotype) is the described species that is phylogenetically closely related to *I. khalidii* (LAH38693, holotype). Interestingly, both species share the uncommon characteristic of having nearly absent caulocystidia. However, in the field, the former can be distinguished by its slightly larger basidiomata (15–35 mm) and dark reddish-brown pileus with appressed radial fibrils. Additionally, *I. rufescens* has reddening flesh. Microscopically, both species can have large basidiospores (10.3 × 5.9 µm for *I. rufescens* and 10.9 × 8.2 µm for *I. khalidii*), but the significantly lower *Q* value (1.75 for *I. rufescens* vs. 1.32 for *I. khalidii*) makes *I. khalidii* basidiospores clearly broadly ellipsoid to ellipsoid in contrast to the more elongated basidiospores of *I. rufescens*. Both species are also distinct ecologically and geographically, as the former was known to occur in litter under *Spyridium globulosum* (Labill.) Benth., *Acacia* Mill., and *Melaleuca* L. shrubs in the heath-scrub habitat of Australia, while the latter is reported for the first time in this study from soil under *Quercus* L. sp. from South Asia (Pakistan) (Matheny and Bougher 2017).

*Inocybe furfurea* (G00053152 lectotype) from France is another phylogenetically relatively close species to *I. khalidii*. Nonetheless, the former can be easily distinguished by its fulvous brown, darker (even nearly black) center, subsquamulose to squamulose cuticle, mouse gray lamellae, and pruinose stipe. Micromorphologically, *I. furfurea* has relatively smaller basidiospores (8.0–10.2 × 5.0–6.0 µm) and often cylindrical pleurocystidia (35–68 × 10–14 µm), while *I. khalidii* has larger basidiospores (7.4–14.3 × 6.1–10.3 μm) and narrowly utriform to lageniform pleurocystidia (37–64 × 11–24 μm) (Kühner 1955). Another phylogenetically allied species to *I. khalidii* is *I. agroterae* (STU: SMNS-STU-F-0901680, holotype) from Germany. Not only molecularly but also macromorphologically, the colors of this species can be strikingly similar to those of *I. khalidii*. Nevertheless, upon close attention, *I. agroterae* exhibits a subsquamulose pileus and a rather pruinose stipe. Microscopically, this species can be separated from *I. khalidii* by the smaller basidiospores (7.1–10.8 × 4.5–6.4 µm) (Bandini et al. 2022b).

*Inocybe catalaunica* (Type SingerIX34, holotype) from Spain is distinct from *I. khalidii* (LAH38693, holotype) by having a tomentose to minutely cracked and subsquamulose brown pileus, a pruinose stipe, relatively smaller and elongated basidiospores (8.3–10.3 × 5–6 µm; Q = 1.7–1.8), and relatively larger pleurocystidia (50–80 × 11–15) (Larsson et al. 2014; Bandini et al. 2022a). *Inocybe rivierana* (STU: SMNS-STU-F-0901249, holotype) from Austria is another orangish genetically close species to *I. khalidii*. Both species can be easily mistaken for each other, as they share subglabrous stages of their pileus cuticles and rather larger basidiospores, which are only slightly broader and smoother in *I. khalidii*. However, the shape of the hymenial cystidia is different, more lageniform–utriform in *I. khalidii* and clearly cylindrical to subcylindrical in *I. rivierana*. Furthermore, the obvious presence of caulocystidia and respective stipe pruinosity sets *I. rivierana* apart (Bandini et al. 2021a).

Several other phylogenetically distinct species in the genus *Inocybe* can be morphologically mistaken for *I. khalidii*. The most obvious case requiring discussion is *I. langei* R. Heim, a famous, vivid, orangish-yellow species with a subglabrous pileus, broadly ellipsoid to ovoid basidiospores, and an equal preference for broad-leaved habitats. In the field, however, a pruinose stipe in the upper half can be observed in *I. langei*, in contrast to *I. khalidii*. Finally, despite the similar spore shape, *I. langei* has much smaller basidiospores (Bandini et al. 2022a). Additional smooth-spored, orangish species worth mentioning are *I. adorabilis*, *I. subbrunnea*, and *I. lindrothii*, for instance, all of which have significantly smaller and more elongated basidiospores, together with the presence of caulocystidia (Larsson et al. 2014; Bandini et al. 2022a).

Additional distant orange species that could be superficially confused with *I. khalidii* are, for instance, the nodulose-spored or marginate-bulbed *I. saliceticola*, *I. bombina*, *I. alnea*, *I. salicis*, *I. caprimulgi*, and *I. lacunarum*. Finally, the genus *Mallocybe* within the family *Inocybaceae* is notorious for its several superficial orangish species, some of which may resemble *I. khalidii*, such as *M. pyrrhopoda*, *M. dulcamara*, and *M. fuscomarginata*, all of which are necropigmented and tomentose together with the other members of the genus *Mallocybe* (Matheny and Bougher 2017; Matheny et al. 2020).

*Inocybe floribundae* is morphologically and phylogenetically distinct within section Marginatae, although it shares affinities with several species bearing nodulose to subnodulose basidiospores and pale brown to yellowish pilei. Species such as *I. diabolica* (JV5712, holotype) (Vauras 1994) and *I. similis* (SF14475, holotype) (Dovana et al. 2020), despite being genetically close, are readily distinguished from *I. floribundae* by their smooth spores. *Inocybe flavobrunnescens* (AH29883, holotype) (Esteve-Raventós et al. 2015) is another yellowish, marginated European taxon with caulocystidia distributed along the stipe, a feature not yet observed in *I. floribundae*, which seems to bear these structures mainly near the apex. *Inocybe corsica* (AH51900, holotype) (Crous et al. 2021) is another phylogenetically related species to *I. floribundae*, yet the two species differ conspicuously in macromorphology. Whereas *I. corsica* typically exhibits yellowish basidiomata with greasy-fibrillose pilei and abundant caulocystidia distributed along the entire stipe, *I. floribundae* possesses beige to brown, distinctly scaly pilei and caulocystidia restricted to the apical region. In addition, the cystidia of *I. floribundae* are obclavate–utriform to subfusiform with short necks, in contrast to the more broadly lageniform to fusiform cystidia of *I. corsica*. The nodules of *I. floribundae* basidiospores are relatively low and blunt (0.7–1.1 µm high), whereas *I. corsica* shows considerably higher and sharper nodules (1.3–2.3 µm high). *I. obtusiuscula* (syn. *I. rufofusca*) (K63A, holotype) (Vauras and Kokkonen 2009) shares a brownish, scaly cap and nodulose basidiospores, but it is an alpine taxon associated with *Salix* in northern Europe, characterized by a distinctly marginate bulb at the stipe base and caulocystidia covering the entire stipe. Similarly, *I. bidumensis* (EL16818, holotype) (Crous et al. 2023) occurs in alpine habitats and shows an ochraceous brown to dark brown, subsquamulose pileus with a wide, darkening basal bulb, a feature absent in *I. floribundae*. Both alpine taxa also differ in basidiospore size and shape, *I. obtusiuscula* with more elongated basidiospores (12.4 × 8.3 µm; Q = 1.49) and *I. bidumensis* with slightly larger and more subheterodiametric spores (10.9 × 7.9 µm; Q = 1.37), in contrast to the broadly subisodiametric basidiospores of *I. floribundae* (10.25 × 7.66 µm; Q = 1.36).

Another phylogenetically close but morphologically distinct species is *I. intricata*, a North American taxon notable for its stellate, asteriform basidiospores (Esteve-Raventós et al. 2015), which are fundamentally different from the bluntly nodulose ornamentation in *I. floribundae*. *Inocybe subrimosa* (Karsten 3223, lectotype) (Esteve-Raventós et al. 2022) is also molecularly related but differs in its more yellowish-brown pileus, a darker stipe that often becomes marginate, and longer, acutely nodulose basidiospores. *Inocybe strickeriana* (KR: KRM0044749) (Bandini et al. 2019) and *I. salicis* (Vauras 2009) possess more reddish-brown pilei, entirely pruinose and marginate stipes, and more protruding spore knobs. The Japanese *I. populea* (TAKK15655, holotype) (Kobayashi and Courtecuisse 2000) can sometimes exhibit subsquamulose pilei and share a similar basidiospore size range, but its crown-shaped, double-nodulate basidiospores differentiate it. Finally, the boreal taxon *I. lacunarum* (JV12244, holotype) (Vauras and Larsson 2016) has a yellower pileus and a marginate stipe and is easily separated by its substellate or spiky spores and by its distinct ecological associations. Additionally, it is geographically separated from other species of the sect. Marginatae s. str., which occur in Europe, North America, and Australia, except for *I. populea* in East Asia (Japan), whereas the new species is endemic to South Asia (Pakistan).

Hence, based on morpho-anatomical comparisons with phylogenetically allied species, we conclude that *I. khalidii* and *I. floribundae* differ from all previously known species of *Inocybe* in morphology, as well as differing from all known ITS, LSU, and *tef*1 sequences. With the addition of these two species, the total number of *Inocybaceae* species reported from Pakistan is increased to 57.

## Supplementary Material

XML Treatment for Inocybe khalidii

XML Treatment for Inocybe floribundae

## Additional information

### Artificial Intelligence (AI) use

The authors accept full responsibility for the content of the manuscript, including the disclosure of any use of AI.

Regarding the use of AI in the preparation of this manuscript, the authors declare the following:

**Description**: Illustrations were produced after tunning four iterations on a large language model (OpenAI 2025), by prompting detailed written taxonomic descriptions together with the upload of a minimum of 2 field photographs per species.

### Author ORCIDs

Sana https://orcid.org/0009-0000-1541-3578

M. Kiran https://orcid.org/0000-0002-3671-9039

F. Rafique https://orcid.org/0009-0008-0377-3419

I. Druzhinina https://orcid.org/0000-0003-2821-5268

V. Fachada https://orcid.org/0000-0002-6575-1943

### Data availability

The accession numbers generated in this study will be available in GenBank after the publication of this article.

## Supplementary materials

Supplementary material 1 ITS + LSU alignment file Data type fas https://binary.pensoft.net/file/1598448This dataset is made available under the Open Database License (http://opendatacommons.org/licenses/odbl/1.0/). The Open Database License (ODbL) is a license agreement intended to allow users to freely share, modify, and use this Dataset while maintaining this same freedom for others, provided that the original source and author(s) are credited. Sana, Munazza Kiran, Frazana Rafique, Irina Druzhinina, Vasco Fachada

Supplementary material 2 Tef file Data type fas https://binary.pensoft.net/file/1598449This dataset is made available under the Open Database License (http://opendatacommons.org/licenses/odbl/1.0/). The Open Database License (ODbL) is a license agreement intended to allow users to freely share, modify, and use this Dataset while maintaining this same freedom for others, provided that the original source and author(s) are credited. Sana, Munazza Kiran, Frazana Rafique, Irina Druzhinina, Vasco Fachada
